# Investigating mu and alpha oscillations as indicators of intra-individual success and inter-individual ability in motor imagery performance

**DOI:** 10.3389/fpsyg.2025.1598196

**Published:** 2025-06-25

**Authors:** María Paula Villabona Orozco, Linn Julie Schwarz, Agnes Hanßen, Cornelia Kranczioch

**Affiliations:** ^1^Neuropsychology Laboratory, Department of Psychology, School of Medicine and Health Sciences, Carl von Ossietzky University of Oldenburg, Oldenburg, Germany; ^2^Neurocognition and Functional Neurorehabilitation Group, Department of Psychology, School of Medicine and Health Sciences, Carl von Ossietzky University of Oldenburg, Oldenburg, Germany

**Keywords:** motor imagery, EEG, mu rhythm, alpha rhythm, imagery ability

## Abstract

Mu and alpha rhythms (8–14 Hz) are recognized for their suppression during motor execution and imagery tasks. Recent research suggests that these oscillations might also serve as a marker of successful motor imagery (MI) performance. This study investigated whether mu and alpha oscillations reflect intra-individual success or inter-individual ability during an MI task, using an adapted methodology consistent with a prior study. EEG data were recorded while young healthy adults (*n* = 19) performed the Test of Ability of Movement Imagery (TAMI). Rhythmic activity was characterized using measures derived from the Better Oscillation Detection Method (BOSC). Contrary to expectations, results do not support the notion that mu/alpha oscillations correlate with imagery success or ability at either the intra- or inter-individual level. However, significant reductions in mu/alpha-wave amplitude were observed during the initiation phase of imagery, underscoring the importance of early neural activity in the MI process, regardless of response success. These findings highlight the intra- and inter-individual variability in mu/alpha rhythms and contribute to the ongoing debate about their role in MI performance.

## 1 Introduction

Motor Imagery (MI), defined as the mental simulation of movements without motor output (Decety, 1996; Sharma et al., 2006), exemplifies how cognitive processes, despite their covert nature, can translate into measurable effects in performance across domains and populations. Since Feltz and Landers’ (1983) seminal work, MI has proven effective in motor performance and skill acquisition, with continued validation over time (Cumming and Ramsey, 2009; Cumming and Williams, 2012; Toth et al., 2020). These benefits extend to neurological rehabilitation, helping stroke patients to relearn movements (Page, 2010; Tong et al., 2017). With the development of MI-based brain computer interfaces, its potential has also broadened in communication and control (Han Yuan and Bin He, 2014; Pfurtscheller et al., 2000; Wolpaw and Wolpaw, 2012).

The wide applicability of MI has been attributed to its shared neural underpinnings with overt motor performance. Brain imaging studies reveal similarities in activation patterns and cognitive planning mechanisms between imagined and executed movements (Lacourse et al., 2005; Lotze et al., 1999; Miller et al., 2010; Sharma and Baron, 2013), supporting the neural simulation of action theory (Jeannerod, 1994, 2001). Electroencephalography (EEG) research has focused on mu rhythm activity. The mu rhythm exhibits a characteristic known as event-related desynchronization (ERD), where its amplitude decreases during the preparation, execution, or imagination of movements (Jasper and Andrews, 1938; McFarland, 2000; Pfurtscheller and Aranibar, 1979). In MI, the mu ERD mirrors the ERD observed during actual movement (Jasper and Penfield, 1949; Neuper and Pfurtscheller, 2010; Pfurtscheller et al., 1997; Pfurtscheller and Neuper, 2006), although it is less pronounced in MI than during motor execution (ME) (McFarland, 2000).

Due to their overlapping frequency range (8–14 Hz), one of the concerns surrounding mu suppression is whether it is distinct from changes in alpha activity (Aleksandrov and Tugin, 2012; Hobson and Bishop, 2016; Larionova et al., 2022). Although often used interchangeably (Fox et al., 2016; Pineda, 2005), they can be distinguished based on factors such as topography and functionality. Examining central and occipital electrodes helps to make this distinction. However, interpreting changes in these regions is difficult due to potential contamination from volume conduction, which can result in bidirectional (mu and alpha) signal mixing (Garakh et al., 2020; Lepage et al., 2008; Tangwiriyasakul et al., 2013). Independent Component Analysis (ICA) offers a solution by separating mixed signals into distinct components, filtering artefacts, and hypothesizing cortical sources (Debener et al., 2010; Onton et al., 2006). While ICA also has its limitations, including the risk that participants do not produce usable mu components (Bowers et al., 2018; Jenson et al., 2020; Nyström, 2008), ICA has successfully differentiated mu and alpha rhythms in mu suppression studies (Moore et al., 2012; Yin et al., 2016).

From a functional perspective, the activation of a specific neural network depends on the MI strategy used, typically categorized as visual or kinesthetic (Decety, 1996; Sharma et al., 2006). Visual imagery involves the mental visualization of the movement and can be experienced from either a first-person (internal) or a third-person (external) perspective (Guillot et al., 2008). Kinesthetic imagery, conversely, involves feeling the sensations associated with the movement (Callow and Hardy, 2004; Guillot and Collet, 2010; Jeannerod, 1995). Neural patterns reflect these strategies: kinesthetic imagery can induce mu power fluctuations linked to sensorimotor cortex activity (Hétu et al., 2013; Stinear et al., 2006), whereas visual imagery may induce alpha rhythm linked to visual processes and attentional engagement (Başar, 2012; Klimesch, 1999; Pfurtscheller, 1992).

Despite the general agreement on the neural overlap between MI and ME, because of MI’s internal nature, the assessment of MI ability remains challenging. Participants may employ different strategies, and individual differences in imagery ability—ranging from athletes (Cumming and Ramsey, 2009; Nakata et al., 2010) to clinical populations (Filippi et al., 2001; Sharma et al., 2006) —can both influence performance and confound results (Kosslyn, 1980, 1994; Richardson, 2020; ter Horst et al., 2013). Objective measures are therefore essential not only to rule out alternative strategies and assess ability, but also to identify individuals who are most likely to benefit from interventions. However, the variety of methods used in movement imagery studies complicates this goal (Chepurova et al., 2022).

The most common assessment methods are self-report questionnaires such as the Movement Imagery Questionnaire (MIQ-3; Williams et al., 2012) and the Kinesthetic and Visual Imagery Questionnaire (KVIQ; Malouin et al., 2007). Here, participants are instructed to imagine a movement and to rate the vividness of the imagery. However, these subjective methods are inherently problematic due to psychological and cognitive biases, such as social desirability and participant’s confidence in their motor imagination skills. The Test of Ability in Movement Imagery (TAMI; Madan and Singhal, 2013) was developed to address these shortcomings. Participants are presented with a series of basic body movements and must select the correct final position from five images. This format introduces both correct and incorrect responses, making the TAMI an objective measure of MI ability.

Correct answers to TAMI questions were found to be associated with reduced mu activity (Chen et al., 2021) particularly during the early stages of the imagery process. This finding was interpreted as evidence that mu activity is involved in indexing MI performance, pointing to mu suppression as a promising objective measure. However, similar results were reported for alpha activity, which also highlights the importance of using methods such as ICA to better disentangle mu and alpha contributions to MI performance.

To date, the relationship between mu-suppression and MI performance is not well understood. In the study by Chen et al. (2021), while successful trials were associated with a more pronounced reduction in the mu band at the individual level, the degree of reduction in mu oscillations did not significantly correlate with overall imagery ability, as measured by the total TAMI score. In the broader field, there is also some heterogeneity regarding the role of mu oscillations in MI performance. Patterns of mu activity may vary depending on factors such as expertise and task complexity. Physical engagement in motor actions has been shown to improve MI, with greater reductions in mu activity reflecting activation of sensorimotor networks, better MI performance, and motor experience (McFarland, 2000; Neuper and Pfurtscheller, 2010; Cannon et al., 2014). While some researchers argue that continuous practice may lead to greater decreases in mu activity (Wriessnegger et al., 2018), the effect of expertise remains unclear across findings from different domains such as music, sport, and rehabilitation (Daeglau et al., 2020; Gibson et al., 2014). These mixed results have been attributed to the inherent variability of neural rhythms in MI studies (Blankertz et al., 2010; Wriessnegger et al., 2020). Further complicating the interpretation is the neural efficiency hypothesis, which proposes that individuals with higher cognitive ability show reduced brain activation during cognitive tasks (Neubauer and Fink, 2009). If sensorimotor engagement is inversely correlated with mu performance, then skilled imagers may show milder reductions in mu activity (Yin et al., 2016).

In sum, MI is linked to a suppression of mu and alpha activity, though the exact nature of this relationship in terms of MI performance remains unclear, given the different assessment methods, variability in neural mechanisms, and MI ability. This study aims to further investigate whether mu and alpha oscillations reflect intra-individual success and/or inter-individual ability in MI performance. To this end, we designed a study drawing on the work and findings of Chen et al. (2021), but with an adapted methodology in which participants completed the TAMI repeatedly to increase the number of trials for mu and alpha analysis. Based on the findings by Chen et al. (2021) we hypothesized the following at the individual level: (1) successful trials would show a more pronounced decrease compared to unsuccessful trials in both motor and visual regions of interest. Each TAMI question consists of four steps, with the initial step corresponding to the onset of MI. During the initial step, (2) we anticipated greater suppression between successful and unsuccessful trials (Aleksandrov and Tugin, 2012; Chen et al., 2021). At the between-subjects level, (3) we expected decreases in mu and alpha activity to reflect motor imagery ability. For the direction of the effect, two possibilities were considered. First, higher scorers in the TAMI might show greater decreases in mu/alpha rhythms for questions with correct answers, indicating higher sensorimotor engagement and ease in performing movements (Cannon et al., 2014; Daeglau et al., 2020). Alternatively, lower scorers on the TAMI may exhibit greater decreases in mu/alpha rhythms for questions with correct answers, indicating greater effort and less neural efficiency in achieving the desired outcome (Neubauer and Fink, 2009).

## 2 Materials and methods

### 2.1 Preregistration and data availability

This study was preregistered on July 10, 2023, before any data examination or analysis. The hypotheses, methods, and analysis plan are available on the Open Science Framework (OSF^1^). The associated project^2^ includes a link to the GitLab repository^3^ which contains raw EEG data in Brain Imaging Data Structure (BIDS; Pernet et al., 2019) format, behavioral data along with EEG analysis and experiment scripts described below.

### 2.2 Participants

The study had a total of 28 participants. Participants were allowed to choose their preferred language for the experiment, with 18 choosing German and 10 English. They were recruited from the student population of the University of Oldenburg through advertisements on the university’s website and word of mouth. Nine participants were excluded: one due to late disclosure of a psychiatric condition, and eight due to missing alpha or mu components. The latter was not part of the preregistered criteria. Participants were excluded if their ICs were not identified, as further analysis would be unfeasible without them. The final sample, therefore, consisted of 19 participants (10 female, aged 21–35 years, M and SD: 27.05 years ± 3.41), all of whom reported normal or corrected-to-normal vision and no psychological or neurological conditions. As tested by a revised version of the Edinburgh Handedness Inventory (rEHI; Veale, 2014), 13 participants were right-handed, five were mixed-handed, and one was left-handed. Since the TAMI is not a lateralized task, we did not expect handedness to influence performance. Before the experiment, participants gave written informed consent. They were reimbursed with €10/h. The experimental procedure was approved by the Commission for Research Impact Assessment and Ethics of the Carl von Ossietzky University of Oldenburg (Drs. EK_22_18).

Given their association with enhanced MI abilities, we also asked participants to report their engagement in sports, instrument playing and video gaming. In the final sample, 11 (6 female) participants reported regular sports engagement, 6 participants played an instrument (4 females) and 4 (2 female) reported playing video games. Five participants did not report on performing any of these activities.

The preregistered sampling plan employed a sequential Bayesian Factor approach as outlined by Schönbrodt and Wagenmakers (2018), with planned sample sizes ranging from a minimum of 15 to a maximum of 25 participants. An initial dataset of 15 participants was collected, of which 12 could be entered into the first data inspection. As moderate evidence was not observed for all confirmatory analyses, recruitment continued as specified in the preregistration. Resources allowed to collect data from three more participants than planned in the preregistration, so the sample was extended to 28 participants. Of the second batch of participants, 6 were excluded from data analysis (see above). The strength of evidence, using Bayes factor (BF) values, followed the criteria established by Lee and Wagenmakers (2014): BF_10_ ≥ 3 indicates moderate evidence for the alternative hypothesis (H_1_) relative to the null (H_0_). BF_01_ ≥ 3 (where BF_01_ = 1/BF_10_) indicates moderate evidence for H_0_ relative to H_1_.

### 2.3 Procedure

Upon arrival at the lab, participants were first given detailed information about the study and asked to provide informed consent. Participants then completed the Kinesthetic and Visual Imagery Questionnaire (KVIQ-10; Malouin et al., 2007), the Motor Imagery Questionnaire (MIQ-3; Williams et al., 2012), and the Block-Tapping Test (BTT; Schellig, 1997). We coded the KVIQ-10 and MIQ-3 into MATLAB scripts (The MathWorks Inc., 2023 version R2023a) to facilitate response coding while following the administration instructions provided in the manuals. Data from the BTT was not analyzed as part of this study. We used the original English versions and German translations (some of them developed for this study) of the questionnaires, depending on the participants’ language of choice. Afterward, participants were instructed to wash their hair to minimize interference from oils or residue, after which the EEG cap was administered.

The main focus of the experiment was the TAMI, the only behavioral measurement taken in conjunction with the EEG recording. The TAMI questionnaire was adapted into a MATLAB script using Psychtoolbox version 3 (Kleiner et al., 2007). While the original TAMI consists of 10 questions, and it is typically administered once — as in the study by Chen et al. (2021) — participants in this study completed TAMI three times, increasing the total number of trials to thirty.

The introduction to the TAMI format began with a practice question. Subsequently, participants carried out the TAMI questionnaire three times, each time in a separate block of the same 10 questions. For the first 10 questions, the original order was followed, but the second and third block of questions were randomly shuffled to prevent recall of sequences from the first block. Each question consists of an initial position, four movement steps and a response page.

**Figure 1:**
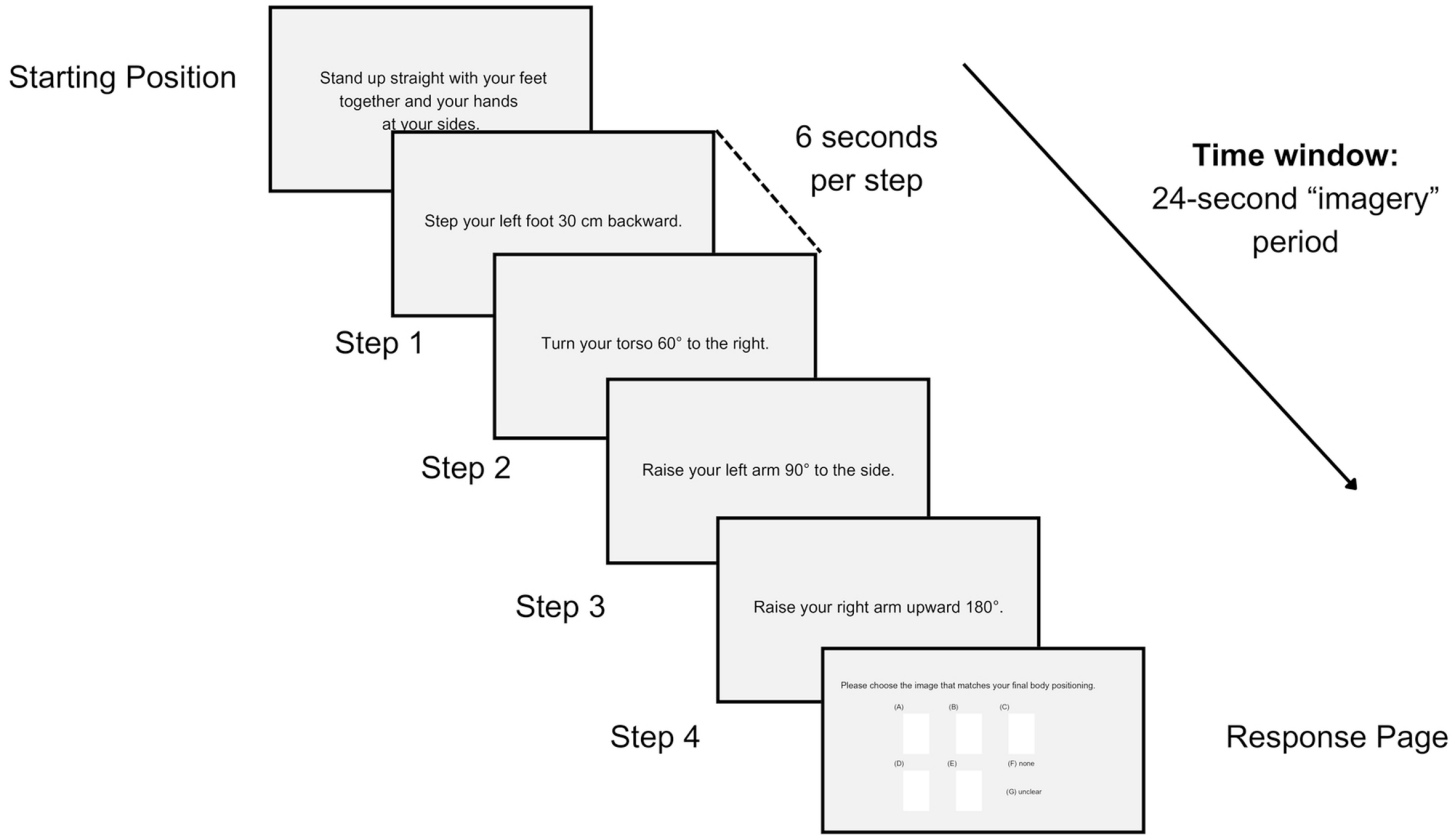
Overview of a single question of the TAMI as seen from the computer screen. Each question involved an initial position, four movement steps (each lasting 6 s), and a response phase, with a 5-s interval between questions.

The TAMI is not a lateralized task; the questions involve randomly combined movement sequences (e.g., head, arm-hand, torso, leg-foot) from both the left and right sides of the body (Madan and Singhal, 2013). Specifically, besides head and torso movements, there are 10 left-sided, 9 right-sided and 4 bilateral movements, totaling 40 movements steps (4 per each question). The movement steps had a duration of 6 s, followed by a 5 s interval between questions (Figure 1). After answering each TAMI question, participants rated its ease or difficulty on a 7-point Likert scale (from very hard to very easy; see section 3 of the Supplementary material). A longer break of 90 s was provided at the end of each block.

**Figure 2:**
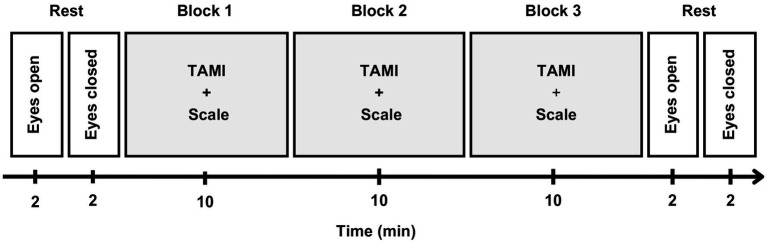
EEG recording timeline. Participants began with 2 min each of eyes-open and eyes-closed resting states. The TAMI questionnaire was presented in three blocks, each block indicated by a light gray background, consisting of 10 randomized questions. After each question, participants rated its difficulty on a 7-point scale. A 90 second break followed each block, and the task ended with a final rest period similar to the initial one.

For the EEG recording, participants sat in an armchair inside a cabin that was soundproofed, electrically shielded, and with dimmed lighting. At the beginning of the computer-based task, a resting state measurement was taken with 2 min eyes open and 2 min eyes closed. This was followed by the TAMI questionnaire blocks (see above) that lasted about 10 min each. The experiment ended with another resting state measurement that mirrored the first. In total, the main experiment lasted about 40 min. At the end of the experiment, participants were given an additional questionnaire asking about their experience and the perspective they took while performing the task. The entire procedure lasted 3 h. Figure 2 illustrates the EEG recording timeline.

### 2.4 Data acquisition

EEG data were recorded using a 64-channel, equidistant infracerebral Ag/AgCl electrode cap (Easycap, Herrsching, Germany) and a BrainAmp EEG amplifier system (BrainProducts, Gilching, Germany). Recording reference was an electrode on the tip of the nose, while a central fronto-polar site served as ground. In addition, two electrodes recorded eye blinks and movements. For the first 11 subjects, data were recorded from a subset of 32 EEG channels. Due to technical issues, all channels were prepared and recorded from subject number 12 onwards, but only the 32-channel subset was used for analysis. The subset corresponds to channel positions where the values were standardized to a theta of 90° for the plane through Fpz, T7, T8 and Oz (Supplementary Table S1). The impedance of the electrodes was kept below 50 kΩ. The sampling rate of EEG signals was 1,000 Hz.

### 2.5 Behavioral analysis

Behavioral measures were derived from the TAMI. A trial was considered successful if the participant gave a correct answer to a TAMI question, whereas a trial was considered unsuccessful if the participant gave an incorrect or “unclear” answer. As our participants belonged to a young and healthy population, we used the TAMI weighted scoring method (Madan and Singhal, 2014), which incorporates weights to ensure statistical sensitivity in identifying higher-scoring individuals. The maximum possible score for this scoring method is 24.

An additional measure of self-perceived motor imagery performance (Ease of Imagination scale) was included. After each TAMI question, participants were asked to rate the ease or difficulty they experienced in completing the task on a scale of 1 to 7, with 1 representing ‘very difficult’ and 7 representing ‘very easy’. The maximum total score per TAMI administration was 70.

Each participant completed the TAMI three times (once per block), allowing us to calculate three separate scores for both the TAMI and Ease of Imagination scale. TAMI scores were calculated by summing the weighted scores of the correct answers within each block. Similarly, Ease of Imagination scale scores were summed within each block. For analyses, both measures were then averaged across the three blocks for each participant.

### 2.6 EEG and spectral analysis

**Figure 3:**
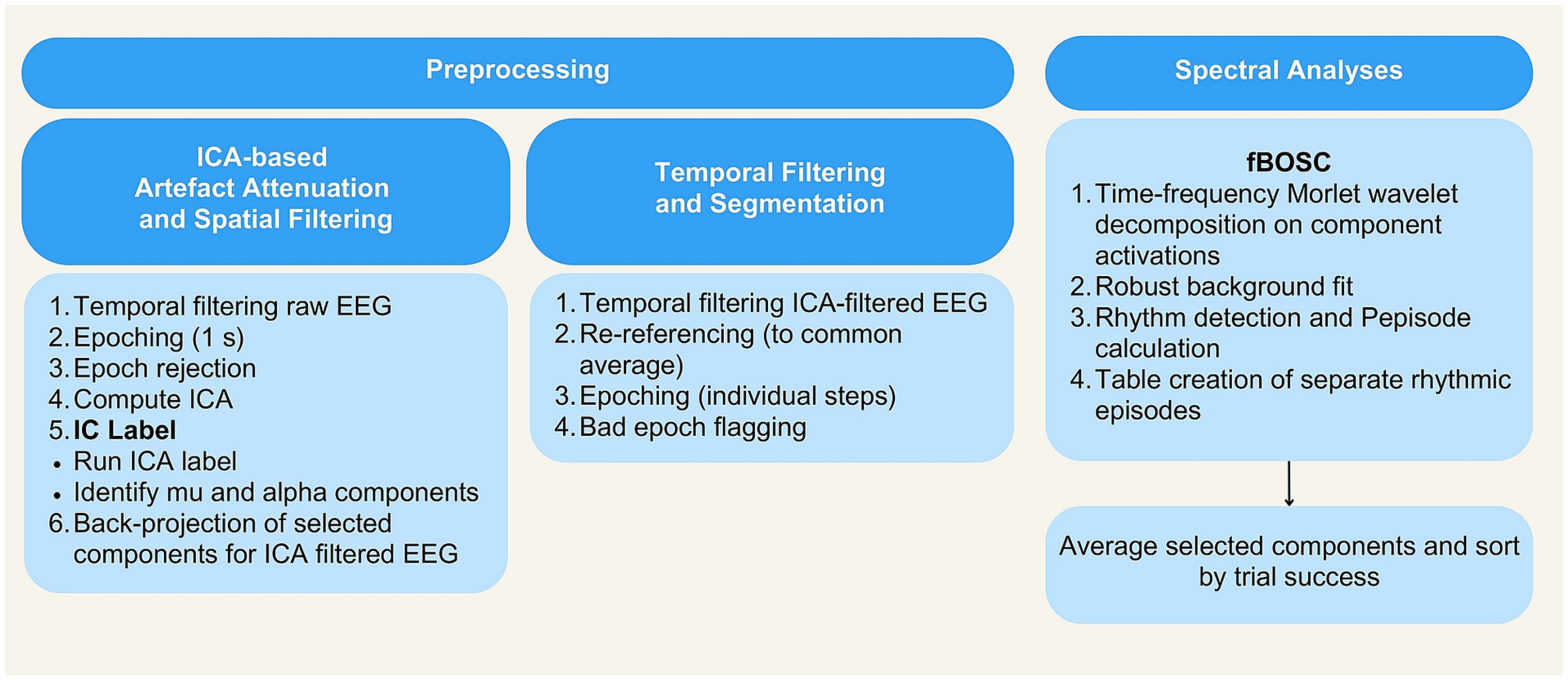
Schematic illustration of the processing pipeline. EEG and spectral analysis.

All EEG data processing steps were scripted and executed in MATLAB (version R2023a, The MathWorks Inc., 2023) and the EEGLAB toolbox (version 2022.0, Delorme and Makeig, 2004). The preprocessing and spectral analysis pipeline is illustrated in Figure 3.

#### 2.6.1 Preprocessing

##### 2.6.1.1 Independent component analysis for artefact attenuation and spatial filtering

A copy of the EEG data was low-pass filtered (windowed sinc FIR filter, cutoff frequency 40 Hz, filter order 310), downsampled to 250 Hz, and then high-pass filtered (windowed sinc FIR filter, cutoff frequency 1 Hz, filter order 776). The data were segmented into consecutive one-second epochs. Segments containing artefacts were excluded using an EEGLAB function (pop_jointprob.m, SD = 3). The remaining data were processed using the extended infomax algorithm to estimate the unmixing weights of 32 independent components. The ICA weights derived from this process were then applied to the original, unfiltered continuous dataset to facilitate paradigm-specific preprocessing (see below). Note that the one-second segmentation was solely used for ICA weight identification, and did not influence later segmentation steps (Stropahl et al., 2018).

Then, the EEGlab plugin ICLabel (Pion-Tonachini et al., 2019) was used to semi-automatically identify independent components (ICs) representing brain activity^4^. ICLabel is a robust and efficient tool having been trained on a large data set with over 200,000 ICs, encompassing a wide variety of experimental designs, recording environments and EEG systems. The classifier uses a combination of spatial, temporal and spectral features to assign probabilistic labels to ICs across multiple categories. ICs classified with high probability as “Brain” were retained for further inspection.

One of the authors (MPVO) and an external expert (S. Debener) identified mu and alpha components. These components were identified based on a heuristic combining topography, spectral content and task modulation. The first two steps of the heuristic consisted of identifying a peak in the traditional power spectrum in the 8–14 Hz range and a distinct dipole-like topography in occipital sites for alpha and sensorimotor sites for mu. When alpha components were not distinct, the topographic distribution between the resting condition of eyes closed and eyes open was compared (Kuhlman, 1978).

The number of mu and alpha components varied according to the participant’s ICA decomposition. Similar to previous studies (Bowers et al., 2018; Jenson et al., 2020; Nyström, 2008), not all participants contributed usable components to the group analysis (*n* = 8), resulting in their subsequent exclusion from further analyses. For included participants, at least one alpha component and one mu component were identified. Selected independent components were retained from each continuous EEG dataset. These components were back-projected to the sensor space, creating data spatially filtered by ICA. For subsequent analyses (see below), when multiple components were found within the same region (e.g., two mu components), their values were averaged.

##### 2.6.1.2 Temporal filtering and segmentation

The spatially filtered data were temporally filtered with a low pass filter (windowed sinc FIR filter, cutoff frequency 40 Hz, filter order 310) and a high pass filter (windowed sinc FIR filter, cutoff frequency 1 Hz, filter order 776). Before applying the high-pass filter, the data were downsampled to 250 Hz to reduce the computational load.

The data were re-referenced to common average and segmented into individual steps of each TAMI question. Each question comprised four sequential steps, each lasting 6 s, for a total duration of 24 s per question. This step-based segmentation approach resulted in 120 epochs per participant (30 TAMI questions * 4 steps). Segmentation was guided by trigger information to identify the onset and offset of each step. We did not include the time period during which participants selected their responses in this analysis, as the focus was on the process of MI rather than the end result (Chen et al., 2021). Remaining artefact-affected epochs not addressed by ICA were identified. Rather than removing these epochs and reducing steps per participant, we retained and flagged them. Only three participants exhibited artefacts, affecting 2–4 segments each.

#### 2.6.2 Spectral analysis

For the preprocessed data, rhythmic activity was characterized using power and Pepisode measures derived from the Better Oscillation Detection Method (BOSC; Whitten et al., 2011). The BOSC method is known for its ability to accurately detect rhythmic activity while minimizing the effects of transient voltage fluctuations (Chen and Caplan, 2017). The duration measure (Pepisode) provided by this approach indicates the presence of oscillations at the selected frequency during a given trial or time segment. For a more complete understanding of the signal, it is recommended to integrate the duration measure with the traditional power measure of the oscillations (Chen et al., 2021; for a comparison of methods, van Vugt et al., 2007). Although Chen’s original study in 2021 focused on the analysis of specific electrodes, the BOSC method has been validated for the detection of oscillations in ICs as used here (Whitten et al., 2011).

To apply the method, we used an openly available tool known as fBOSC^5^. The tool incorporates a modification of the 1/f fitting procedure using the ‘fitting oscillations and one over f’ (FOOOF) spectral parameterization algorithm (Donoghue et al., 2020). Unlike previous approaches within the BOSC framework, the fBOSC demonstrates improved sensitivity, reduced false alarm rate, resilience to various noise sources, and offers a user-friendly implementation (Seymour et al., 2022).

Spectral analysis was performed on mu and alpha IC activations. Each segment of the IC activations was time-frequency decomposed using Morlet wavelets with a width of six cycles. We used frequency sampling at logarithmically spaced frequencies (log base 2) between 2 and 41 Hz. Power measurements were then calculated by squaring the instantaneous amplitude of the complex convolution results, then log-transformed and normalized, dividing by the mean log power derived from the entire recording session for each frequency band and IC. After the wavelet transform, 0.5 s were removed from both the beginning and end of the initial time-frequency matrix to avoid potential artefacts arising from the convolution. Following the fBOSC approach, 0.5 s were also removed after episode detection and during background estimation. Notably, the pruned time points occurred at the very start and end of the instruction-reading period, intervals during which little motor imagery activity was expected, thereby making our analyses more specific.

The power threshold was set at the 99th percentile of the power values in a chi-squared distribution for each frequency. A duration threshold of three oscillation cycles was also applied. The parameters chosen were based on both the original study and the recent fBOSC implementation validated also in alpha oscillations (Seymour et al., 2022). After applying the BOSC method, when more than one alpha or mu IC was present, they were averaged. Specifically, from the output of the fBOSC implementation, which is 3-dimensional (component, trials, frequency bins), we first sorted the data according to conditions (i.e., successful and unsuccessful trials within a given participant). Then, for each condition, we averaged the values across trials for each component. Finally, if multiple components were present within a domain (mu or alpha), we averaged the results across components to obtain a single, representative measure per participant and condition. Each participant completed the TAMI three times, contributing between 2 and 23 unsuccessful trials, with an average of 8 unsuccessful trials per participant.

### 2.7 Statistical analysis

For confirmatory hypotheses, Bayesian hypothesis testing was the primary inferential framework used to determine whether the observed data were more consistent with the alternative hypothesis (Bayes factor BF_10_ > 1) or the null hypothesis (BF_01_ > 1, where BF_01_ = 1/BF_10_). Confirmatory analyses were conducted in JASP statistical software with default prior settings (version 0.17.1.0; JASP Team, 2023). Bayesian inference was chosen over frequentist analysis, contrary to Chen et al. (2021) study, due to practical and conceptual advantages. First, the expected sample size was constrained by a two-month data collection window, which represented about half of the original study (*n* = 57). The sequential Bayesian design allowed us to address potential power limitations while preserving interpretive flexibility and statistical validity. Even when the threshold for moderate evidence is not reached, the direction and strength of BFs remain interpretable (Schönbrodt and Wagenmakers, 2018). Importantly, Bayesian Hypothesis enables researchers to distinguish between absence of evidence and evidence of absence. Non-significant *p*-values may result from low statistical power rather than support for the null hypothesis. BFs, by contrast, provide a graded measure of evidence offering insights into whether the data meaningfully support the null, the alternative or remain inconclusive (Keysers et al., 2020).

Additionally, we conducted an exploratory analysis using the rmcorr package in R (Bakdash and Marusich, 2017) and its web/standalone Shiny application (Marusich and Bakdash, 2021). In these analyses, a *p*-value of 0.05 was used to test the significance of the null hypothesis. We relied solely on *p*-values here because Bayesian methods are not currently available for this package.

Because of the sequential Bayesian Factor design, we monitored results as data accumulated. Thus, statistical tests were performed twice: after collecting data from the first batch of participants (which provided inconclusive evidence for either hypothesis) and later after the full sample was collected. The interim analysis of the first batch, affected the assumptions of frequentist methods applied in the exploratory analysis. However, the results, interpretations, and conclusions are based exclusively on Bayes factors. For transparency and comparison with previous work, frequentist analyses of the confirmatory analyses (conducted only on the full sample), are also provided in the Supplementary Tables S6–S8. Additional frequentist results are also provided in some exploratory analyses for comparison, but this did not guide our inference.

#### 2.7.1 Motor imagery success

To investigate whether mu/alpha oscillations reflect intra-individual success, we tested our first and second hypotheses using a three-way Bayesian repeated measures analysis of variance (rm-ANOVA). The dependent variable was the mu/alpha oscillation measure (power or Pepisode) and we conducted separate analyses for each of them. The independent variables were MI success (correct/incorrect answers to a TAMI question), region (motor/visual ICs), and movement steps of the TAMI questions (1, 2, 3, 4). As mentioned earlier, for participants with multiple alpha or mu components, values were averaged for all further analyses. Hypothesis 1 tested the main effect of ‘Success’, while Hypothesis 2 tested the interaction between ‘Success’ and ‘Step’, consistent with what Chen and colleagues found in their study (2021).

#### 2.7.2 Motor imagery ability

To explore whether mu/alpha oscillations indexed inter-individual ability, we tested our third hypothesis with a Bayesian Pearson correlation. Hypothesis 3 correlations were performed between the average TAMI weighted score across three blocks and the difference in mu/alpha oscillation suppression (successful – unsuccessful). This measure of difference was chosen following the analysis in Chen’s study, who argued that it could be used to infer the ability to suppress mu/alpha oscillations and MI ability. This correlation was performed once for power and once for Pepisode measures.

#### 2.7.3 Pre-registered exploratory analyses

In relation to Hypothesis 3, as Chen et al. (2021) did not find a significant relationship between mu/alpha oscillatory activity and MI ability measured by the TAMI, we decided to examine this relationship with another measure, ease of imagination. Therefore, an additional Bayesian Pearson correlation was performed between the average Ease of Imagination scale score over three blocks and the difference in mu/alpha oscillation suppression. Again, this correlation was performed once for power and once for Pepisode measures.

For the main correlation analyses, performance scores and oscillation data were averaged across the three repetitions of the TAMI. To explore whether this might obscure a possible correlation between measures, we performed a repeated measures correlation (Bakdash and Marusich, 2017). In brief, repeated measures correlation (rmcorr) identifies common within-individual associations across occasions while maintaining assumption of independence, thus avoiding bias from aggregated data. This method provides strong statistical power by estimating a common regression slope without the need for averaging. First, we performed a correlation between the weighted TAMI score and the average measures of mu/alpha oscillations (power and Pepisode) for each block. And second, we performed a correlation between the Ease of Imagination scale scores and the average mu/alpha oscillation measures (power and Pepisode), also for each block.

## 3 Results

### 3.1 Behavioral results

**Figure 4:**
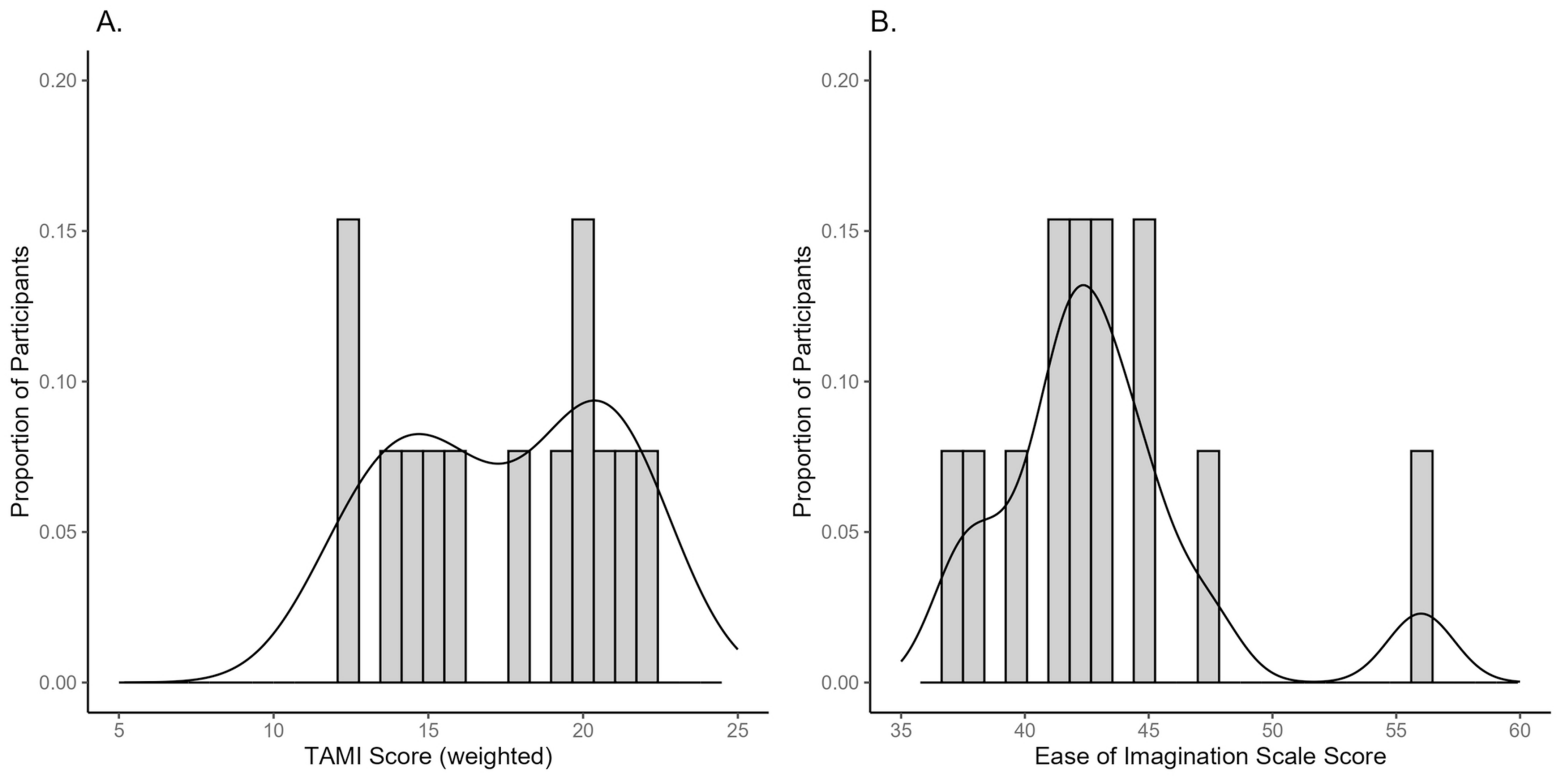
Distribution of participants’ scores on **(A)** the TAMI, and **(B)** the Ease of Imagination scale.

Participants’ performance on the TAMI was scored based on the weighted sum of the correct answers within each block, resulting in three total scores per participant, which were then averaged. The Ease of Imagination scale performance was also scored by summing their ratings within each block and then averaging across blocks. For the TAMI, the mean score was 15.93 (SD = 4.28) out of 24 points and for the Ease of Imagination scale it was 43.47 (SD = 6.82), out of a possible 70 points. Figure 4 shows the distribution of TAMI and the Ease of Imagination scale scores. TAMI scores are normally distributed but slightly skewed to the right, which is consistent with previous studies (Madan and Singhal, 2014). In contrast, the Ease of Imagination scale scores were left-skewed (skewness of 1.076, *p* = 0.021). Full descriptive statistics can be found in the Supplementary Table S2.

To ensure that handedness was not a confounding variable in the performance of the TAMI, we conducted additional correlations between EHI handedness and TAMI scores for each block. Results revealed weak, negative correlations for block 1 (*r* = −0.190, BF₁₀ = 0.377, *p* = 0.436) and block 2 (*r* = −0.105, BF₁₀ = 0.309, *p* = 0.670) and a very small positive correlation for block 3 (*r* = 0.029, BF₁₀ = 0.286, *p* = 0.906). All BFs resulting from Bayes correlations were below 1, indicating moderate evidence for the null hypothesis of no relationship between handedness and TAMI performance, which was in correspondence with the non-significant frequentist correlations.

### 3.2 Electrophysiological data

#### 3.2.1 Motor imagery success

**Figure 5:**
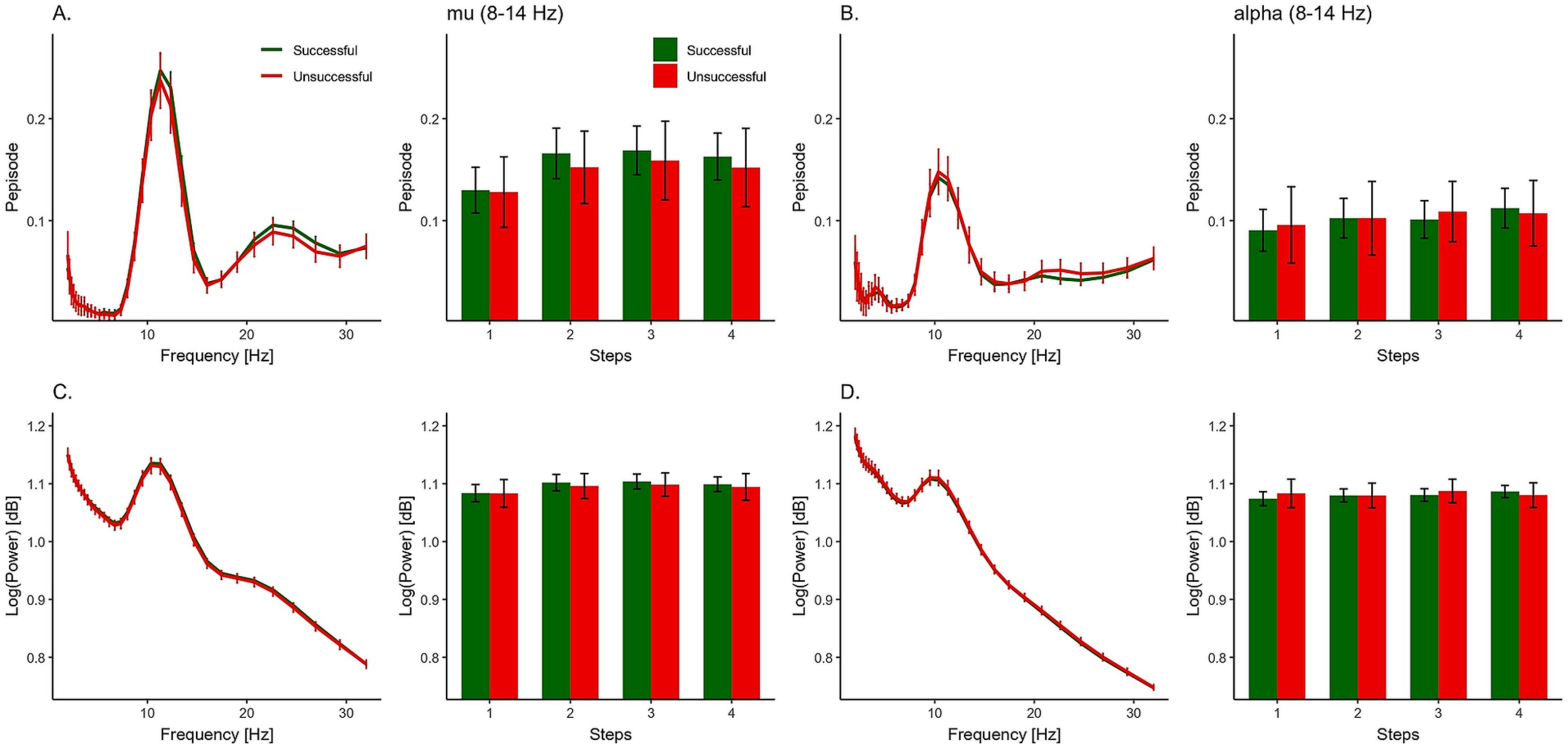
Oscillatory activity and MI success. Group-level comparison of neural oscillations during successful (green) and unsuccessful (red) trials. Pepisode measurements for mu **(A)** and alpha **(B)** bands are shown across movement steps 1–4, alongside their corresponding log-transformed power spectra **(C,D)**. Error bars indicate standard error of the mean (SEM).

We tested the effect of successful MI performance (Success*) on mu and alpha oscillations by comparing successful and unsuccessful responses on the TAMI. Figure 5 and Table 1 show the summary statistics for the oscillation measurements, including both Pepisode and power. The Bayesian rm-ANOVA provided moderate evidence against the effect of ‘Success’ for both the Pepisode (BF_incl_ = 0.264) and power (BF_incl_ = 0.193) measures. In addition, there was limited evidence for a main effect of region on either measure (refer to Table 2 for a full analysis of effects).

**Table 1:** Summary statistics for mu activity using Pepisode and log power [dB] measures.

Success	Region	Step	Pepisode			Log (Power) [dB]		
			Mean	SD	SE	Mean	SD	SE
Successful	Visual	Step 1	0.091	0.084	0.019	1.074	0.049	0.011
		Step 2	0.102	0.099	0.023	1.080	0.054	0.012
		Step 3	0.101	0.114	0.026	1.080	0.067	0.015
		Step 4	0.112	0.121	0.028	1.087	0.068	0.016
	Motor	Step 1	0.130	0.088	0.020	1.084	0.052	0.012
		Step 2	0.166	0.110	0.025	1.102	0.058	0.013
		Step 3	0.169	0.116	0.027	1.104	0.060	0.014
		Step 4	0.163	0.109	0.025	1.099	0.059	0.014
Unsuccessful	Visual	Step 1	0.096	0.094	0.021	1.083	0.056	0.013
		Step 2	0.102	0.107	0.025	1.080	0.055	0.013
		Step 3	0.109	0.130	0.030	1.087	0.075	0.017
		Step 4	0.107	0.101	0.023	1.080	0.057	0.013
	Motor	Step 1	0.128	0.084	0.019	1.083	0.050	0.011
		Step 2	0.152	0.115	0.026	1.096	0.064	0.015
		Step 3	0.159	0.103	0.024	1.098	0.059	0.014
		Step 4	0.152	0.109	0.025	1.094	0.055	0.013

**Table 2:** Analysis of effects Pepisode and log power [dB] measures.

Effects	Pepisode				Log (Power) [dB]			
	P(incl)	P(incl|data)	BF_incl_	BF_excl_	P(incl)	P(incl|data)	BF_incl_	BF_excl_
Success	0.263	0.188	0.264	3.788	0.263	0.154	0.193	5.181
Region	0.263	0.348	0.702	1.425	0.263	0.395	0.786	1.272
Step	0.263	0.748	5.255	0.190	0.263	0.422	0.850	1.176
Success ✻ Region	0.263	0.081	1.026	0.975	0.263	0.039	0.476	2.101
Success ✻ Step	0.263	0.023	0.103	9.709	0.263	0.015	0.174	5.747
Region ✻ Step	0.263	0.091	0.253	3.953	0.263	0.069	0.319	3.135
Success ✻ Region ✻ Step	0.053	1.885 × 10^−4^	0.106	9.434	0.053	1.585 × 10^−4^	0.187	5.348

The BF_incl_ can be interpreted as evidence in the data for including a predictor. For interpretation, BF_excl_ was given with BF_excl_ = 1/BF_incl_. The analysis of effects compares models that contain the effect to equivalent models stripped of the effect. Analysis suggested by Sebastiaan Mathôt (van den Bergh et al., 2020).

For our second hypothesis, examining changes in oscillations over time, we expected an interaction between ‘Success’ and ‘Step’. However, the analysis showed moderate evidence against this effect for Pepisode (BF_incl_ = 0.103) and power measures (BF_incl_ = 0.174). For Pepisode measures, however, ‘Step’ was included as a predictor in the best performing model (Supplementary Table S3). There was a main effect of ‘Step’ (Table 2), indicating that the likelihood of data occurrence in models with ‘Step’ as a predictor was approximately 5.255 times higher than those without. However, according to power measures, there was only anecdotal evidence against the effect of ‘Step’ (BF_incl_ = 0.850).

**Figure 6:**
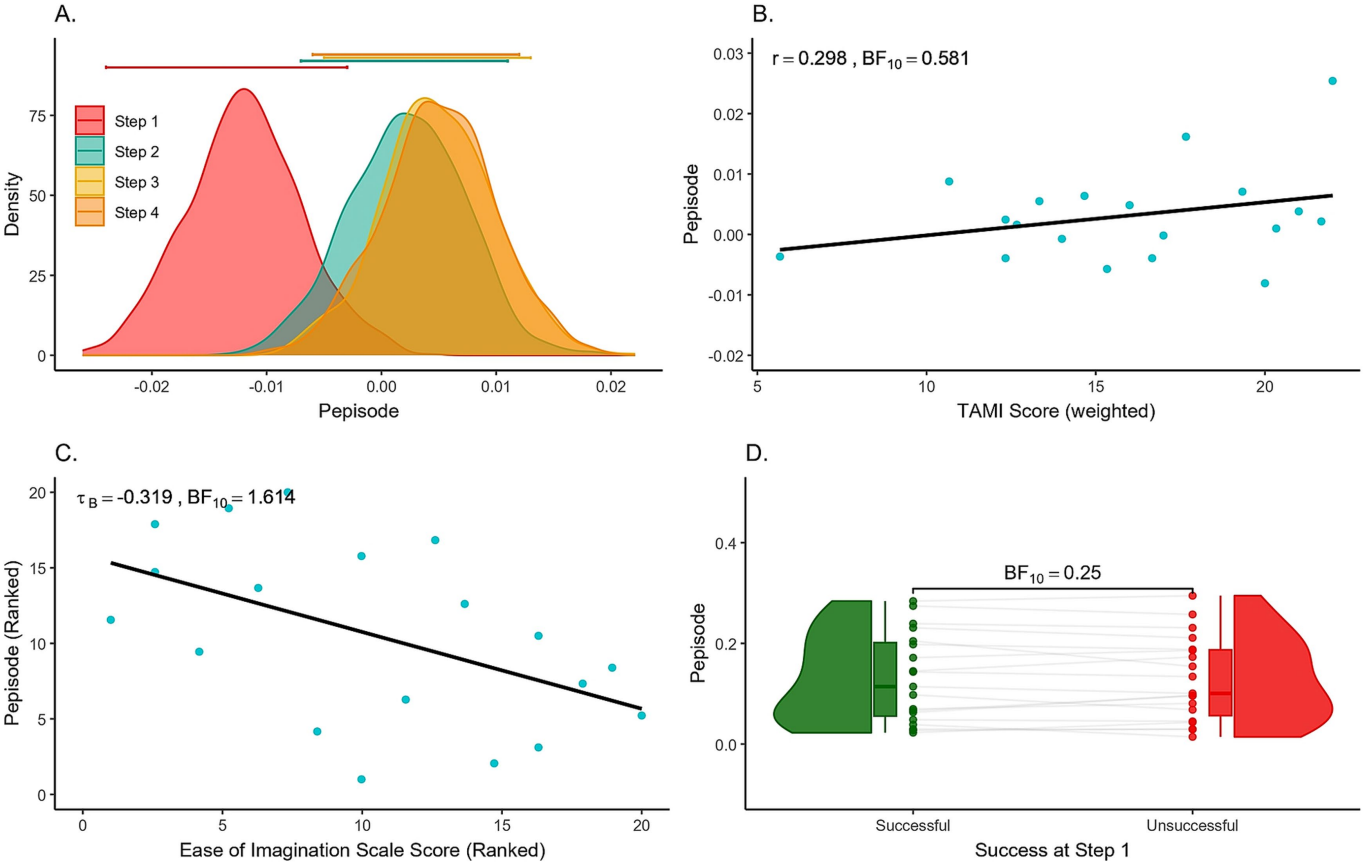
**(A)** Posterior distributions of the effect of each Step in Pepisode measures. Error bars above each density represent 95% credible intervals. **(B)** Motor Imagery Ability vs. TAMI Scores. Scatter plot showing participants’ size of motor imagery success (successful - unsuccessful trials) using Pepisode measures from the motor region, compared to their TAMI scores. Each dot represents one participant. **(C)** Motor imagery ability vs. Ease of Imagination scores. Scatter plot showing participants’ size of motor imagery success (successful - unsuccessful trials) using Pepisode measures from the motor region, compared to their Ease of Imagination scores. Each dot represents one participant. Both axes show ranked data for tau B correlation. **(D)** Raincloud plot of Success at Step 1 showing Pepisode values in the motor region. Each dot represents one participant, with overlaid box plots indicating the interquartile range and median. The density distribution illustrates data spread.

To understand which levels of ‘Step’ differ from each other, we conducted a *post hoc* test on this predictor (Table 3). The adjusted posterior odds show (1) strong evidence (odds of about 24) that Pepisode measures differ between Step 1 and Step 2, (2) moderate evidence (odds of about 8) that Pepisode measures differ between Step 1 and Step 3, and (3) strong evidence that Pepisode measures differ between Step 1 and Step 4 (odds of about 29). On the other hand, we found (4) moderate evidence (odds of 0.150, 0.146, 0.129) that Pepisode measures of Step 2 and Step 3, Step 2 and Step 4, and Step 3 and Step 4 are the same. The magnitude of each ‘Step’ on the Pepisode measures are displayed through the model averaged posteriors in Figure 6A, highlighting how Step 1 has lower Pepisode values than all other steps. The parameter estimates of the marginal posterior effects are shown in Supplementary Table S5.

**Table 3:** *Post hoc* test for step factor from Pepisode measures.

		Prior odds	Posterior odds	BF_10, U_	Error %
Step 1	Step 2	0.414	24.096	58.173	2.804 × 10^−8^
	Step 3	0.414	8.362	20.188	9.813 × 10^−8^
	Step 4	0.414	28.968	69.934	2.240 × 10^−8^
Step 2	Step 3	0.414	0.062	0.150	0.097
	Step 4	0.414	0.060	0.146	0.099
Step 3	Step 4	0.414	0.053	0.129	0.108

The first two columns show the steps being compared, while the third and fourth columns show the adjusted prior and posterior model odds. The posterior odds have been corrected for multiple testing by fixing to 0.5 the prior probability that the null hypothesis holds across all comparisons (Westfall et al., 1997). The fifth column shows the uncorrected Bayes factor supporting the alternative hypotheses of different magnitudes. The last column shows the numerical error in the calculation of the Bayes factor. Individual comparisons are based on the default *t*-test with a Cauchy (0, *r* = 1/sqrt(2)) prior.

#### 3.2.2 Motor imagery ability

We investigated whether mu or alpha suppression could indicate individual MI ability, by performing Bayesian Pearson correlations. First, we correlated the difference in mu oscillation suppression (successful – unsuccessful) with weighted TAMI scores across participants, and then with scores on the Ease of Imagination scale. Scatter plots illustrating these relationships for the mu oscillations and power measures can be found in Figures 6B,C. The complete correlation matrix is available in Table 4, and the full figures can be accessed in the Supplementary Figures S1, S2.

**Table 4:** Bayesian and repeated measures correlations for relationships between mu and alpha oscillatory activity and motor imagery ability.

Score	Comparison	Pearson		Kendall		rmcorr	
		*r*	BF₁₀	Tau B	BF₁₀	r_rm_(37)	*p*
TAMI (weighted)	Ease of Imagination scale	0.027	0.285	0.290	1.204	0.369	0.021*
	Pepisode mu	0.298	0.581	0.117	0.369	0.020	0.920
	Pepisode alpha	−0.128	0.322	−0.070	0.318	−0.280	0.079
	Power mu	0.288	0.552	0.152	0.433	0.010	0.945
	Power alpha	−0.498	2.553	−0.375	3.124	−0.320	0.046*
Ease of imagination scale	Pepisode mu	−0.389	1.006	−0.319	1.614	−0.210	0.203
	Pepisode alpha	0.189	0.376	0.224	0.682	−0.210	0.197
	Power mu	−0.307	0.609	−0.177	0.496	−0.180	0.276
	Power alpha	−0.075	0.296	−0.024	0.295	−0.240	0.148

TAMIw score and Ease of Imagination scale compared with the Pepisode and log power [dB] values. Pepisode and log power values indicate the difference between successful and unsuccessful trials on the TAMI and Ease of Imagination scale scores. For Bayesian analysis, scores are averaged over the three blocks per participant. For repeated measures, scores are considered separately for each block. *p-values ≤ 0.05.

Using a two-sided alternative hypothesis, mu suppression differences were positively correlated with the TAMI score in both Pepisode (*r* = 0.298) and power (*r* = 0.288) measures. However, Bayes factors provided inconclusive evidence favoring the null hypothesis (Pepisode, BF_10_ = 0.581; power, BF_10_ = 0.552). In contrast, the difference in alpha suppression was negatively correlated, with moderate evidence supporting null hypothesis for Pepisode (*r* = −0.128, BF_10_ = 0.322), while the evidence for power measures was inconclusive toward the alternative (*r* = −0.498, BF_10_ = 2.533).

For the Ease of Imagination scale, the patterns were reversed. The mu difference measure had a negative relationship, with anecdotal evidence supporting the alternative (Pepisode: tau *B* = −0.319, BF_10_ = 1.614) or the null hypothesis (power: tau *B* = −0.177, BF_10_ = 0.496). Conversely, the alpha difference measure showed a weak positive correlation for Pepisode (tau *B* = 0.224, BF_10_ = 0.682) and a negative correlation for power (tau *B* = −0.024, BF_10_ = 0.295), with anecdotal evidence supporting the null hypothesis for both.

### 3.3 Exploratory analyses

#### 3.3.1 Success at first step

Since mu/alpha oscillation suppression was strongest in the first movement step, we also examined the relationship between mu/alpha oscillations from Step 1 and individual success. As shown in Figure 6D the patterns of individual success showed little difference between unsuccessful and successful trials, with some cases even showing fewer detected oscillations in unsuccessful trials. The strength of evidence ranged from anecdotal to moderate in favor of the null hypothesis. Additional figures including BF values for both regions and measures are available in the Supplementary Figure S3.

#### 3.3.2 Motor imagery ability and ease of imagination

**Figure 7:**
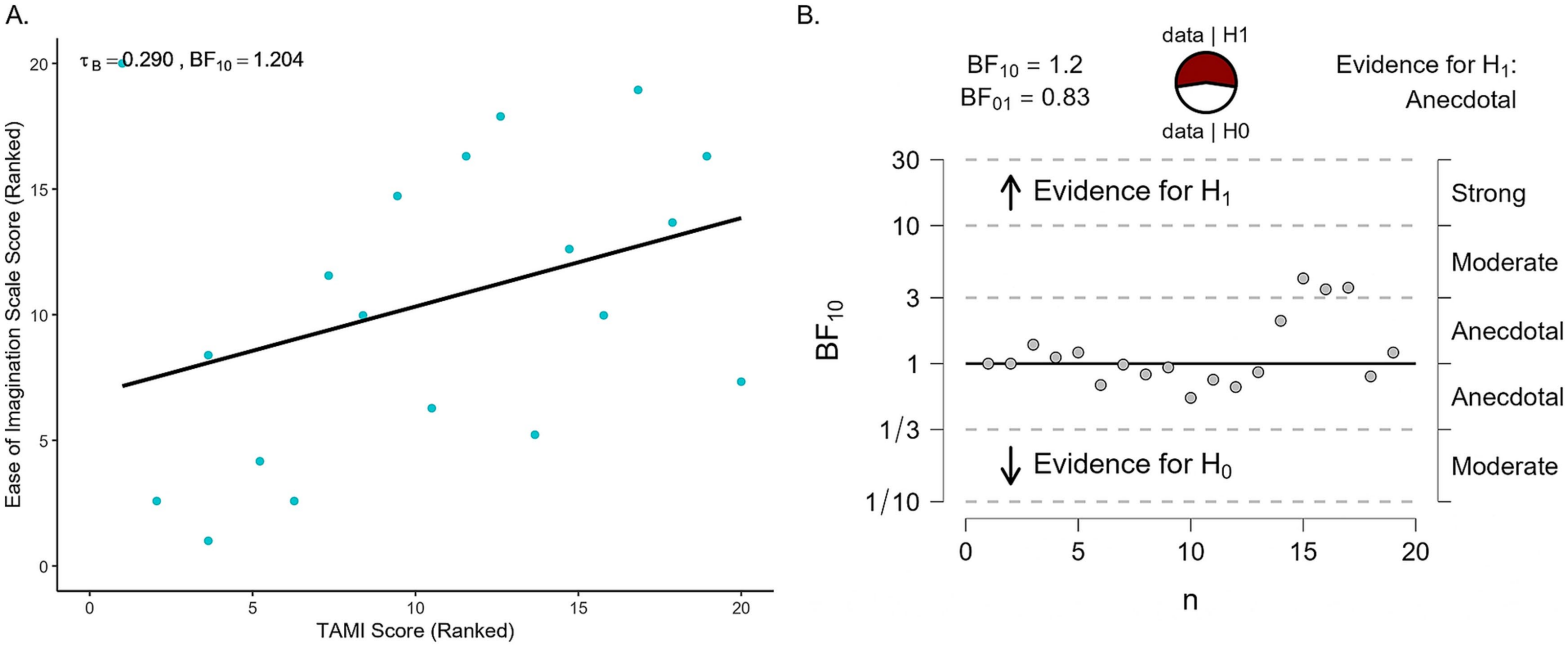
TAMI vs. Ease of Imagination scores. **(A)** Scatter plot showing participants’ TAMI weighted scores compared to their Ease of Imagination scores. Each dot represents one participant. Both axes show ranked data for tau B correlation. **(B)** Sequential development of the evidence as the data accumulates.

As the study included two different measures of MI performance, we examined their relationship with a Bayesian Pearson correlation (Figure 7). This analysis provided inconclusive evidence of a weak positive correlation (*r* = 0.027, BF_10_ = 0.285; tau *B* = 0.290, BF_10_ = 1.204). For illustration, Figure 7 includes the sequential analysis plot showing how the Bayes factor changes with each added data point, with the BF_10_ fluctuating between anecdotal and moderate evidence in support of the alternative hypothesis.

#### 3.3.3 Repeated measures correlations

To account for repeated testing of participants across three blocks, we performed repeated measures correlations (where the block serves as the repeated measure) for each Bayesian Pearson correlation performed in the context of the third hypothesis. A summary is presented in Table 4. For correlations related to the TAMI score, results were consistent in both direction and significance with the Bayesian results. The mu difference measure and the TAMI scores showed positive, but weak and non-significant relationships [Pepisode, *r*_rm_ (37) = 0.020, *p* = 0.920; power, *r*_rm_ (37) = 0.010, *p* = 0.945]. In comparison, the alpha difference measure showed negative, weak correlations [Pepisode, *r*_rm_ (37) = −0.280, *p* = 0.079; power, *r*_rm_ (37) = −0.320, *p* = 0.046], with only the latter reaching statistical significance.

For the Ease of Imagination scale scores, most correlations align with Bayesian results; however, mu and alpha difference measures are negatively correlated, with weak and non-significant associations.

**Figure 8:**
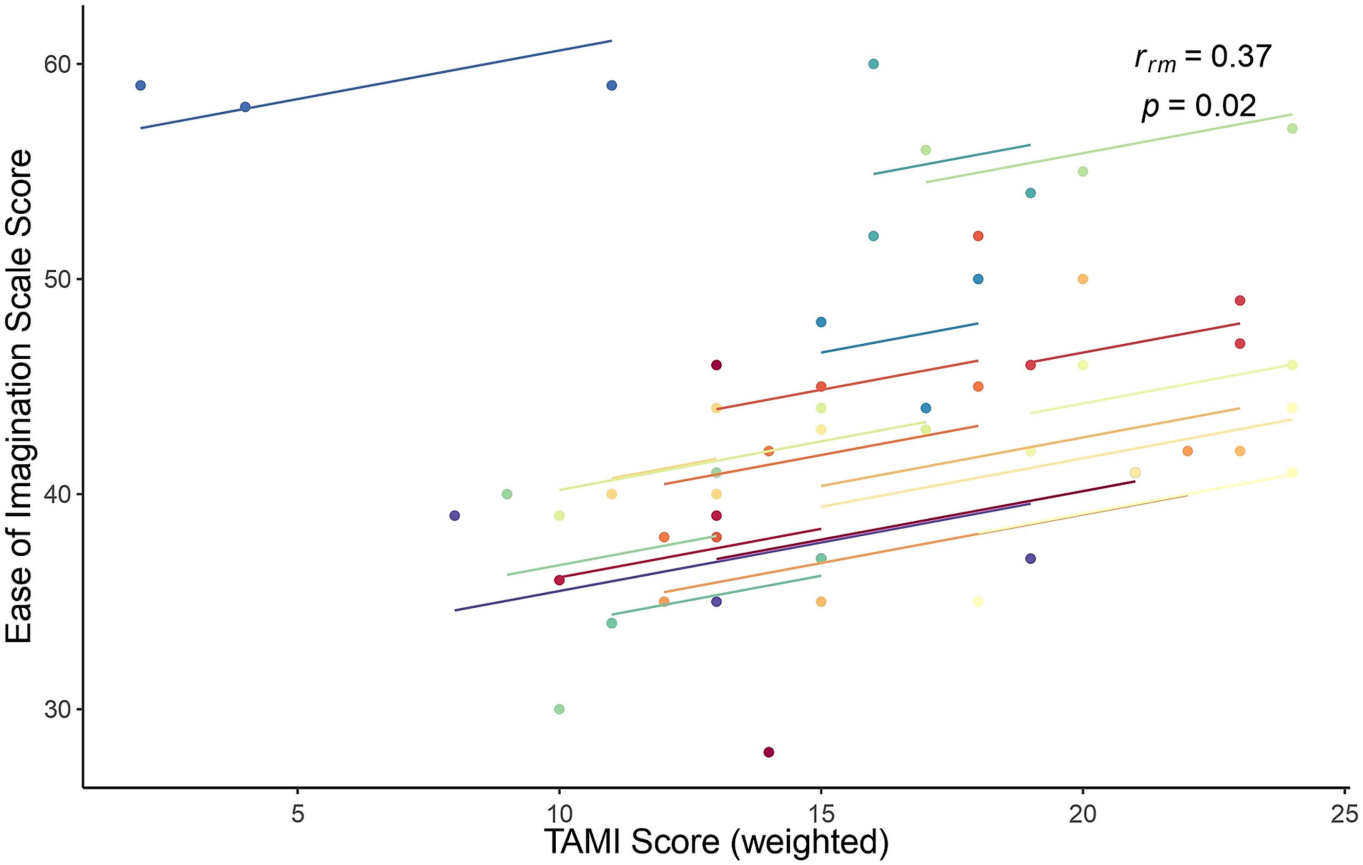
Rmcorr plot for the relationship between the TAMI (weighted) scores and the Ease of Imagination scale scores for each of the three blocks. Each color represents one participant.

Finally, the association between TAMI scores and Ease of Imagination scale scores was similar to the Bayesian correlation: weak [*r*_rm_ (37) = −0.369], but statistically significant (*p* = 0.021). A scatter plot showing this relationship is shown in Figure 8.

#### 3.3.4 Replication Bayes factors

The need to assess and improve reproducibility in science has led to the development of statistical methods to evaluate the extent to which a replication study is successful. The replication Bayes factor is one such method, quantifying the evidence from a direct replication attempt given the data from the original study (Ly et al., 2019). Here, we made use of the replication Bayes factor to assess how the second batch of data influenced the findings from the first batch. To do this, we calculated the replication Bayes factors for our main hypotheses. These were obtained by dividing the Bayes factors from the full dataset (19 subjects) by those from the first batch dataset (13 subjects). Tables 5, 6 provide the results for our three hypotheses. Overall, the evidence from the second batch, informed by the first, did not change substantially in direction or magnitude.

**Table 5:** Replication Bayes factor for Hypothesis 1 and 2.

Measure	Hypothesis	Case	Complete BF₁₀ (dorig, drep)	Original BF₁₀ (dorig)	Replication BF₁₀ (drep)
Pepisode	1	Success	0.264	0.434	0.608
	2	Success ✻ Step	0.103	0.159	0.648
		Step	5.255	3.151	1.668
Log (Power) [dB]	1	Success	0.193	0.284	0.680
	2	Success ✻ Step	0.174	0.118	1.475

Main effect of step was added in the analysis for Pepisode measures as we found moderate evidence. drep = (dorig, drep)/dorig.

**Table 6:** Replication Bayes factor for Hypothesis 3.

Hypothesis 3				
Score		Complete BF₁₀ (dorig, drep)	Original BF₁₀ (dorig)	Replication BF₁₀ (drep)
TAMI (weighted)	- Ease of Imagination scale	1.204	0.868	1.387
	- Pepisode mu	0.581	0.689	0.843
	- Pepisode alpha	0.322	0.359	0.897
	- Power mu	0.552	0.606	0.911
	- Power alpha	2.553	0.795	3.211
Ease of imagination scale	- Pepisode mu	1.614	0.574	2.812
	- Pepisode alpha	0.682	2.851	0.239
	- Power mu	0.496	0.380	1.305
	- Power alpha	0.295	0.574	0.514

For correlations that included the Ease of Imagination scale, the tau B Bayes factor instead of Pearson’s was considered. drep = (dorig, drep)/dorig.

#### 3.3.5 Topographies

**Figure 9:**
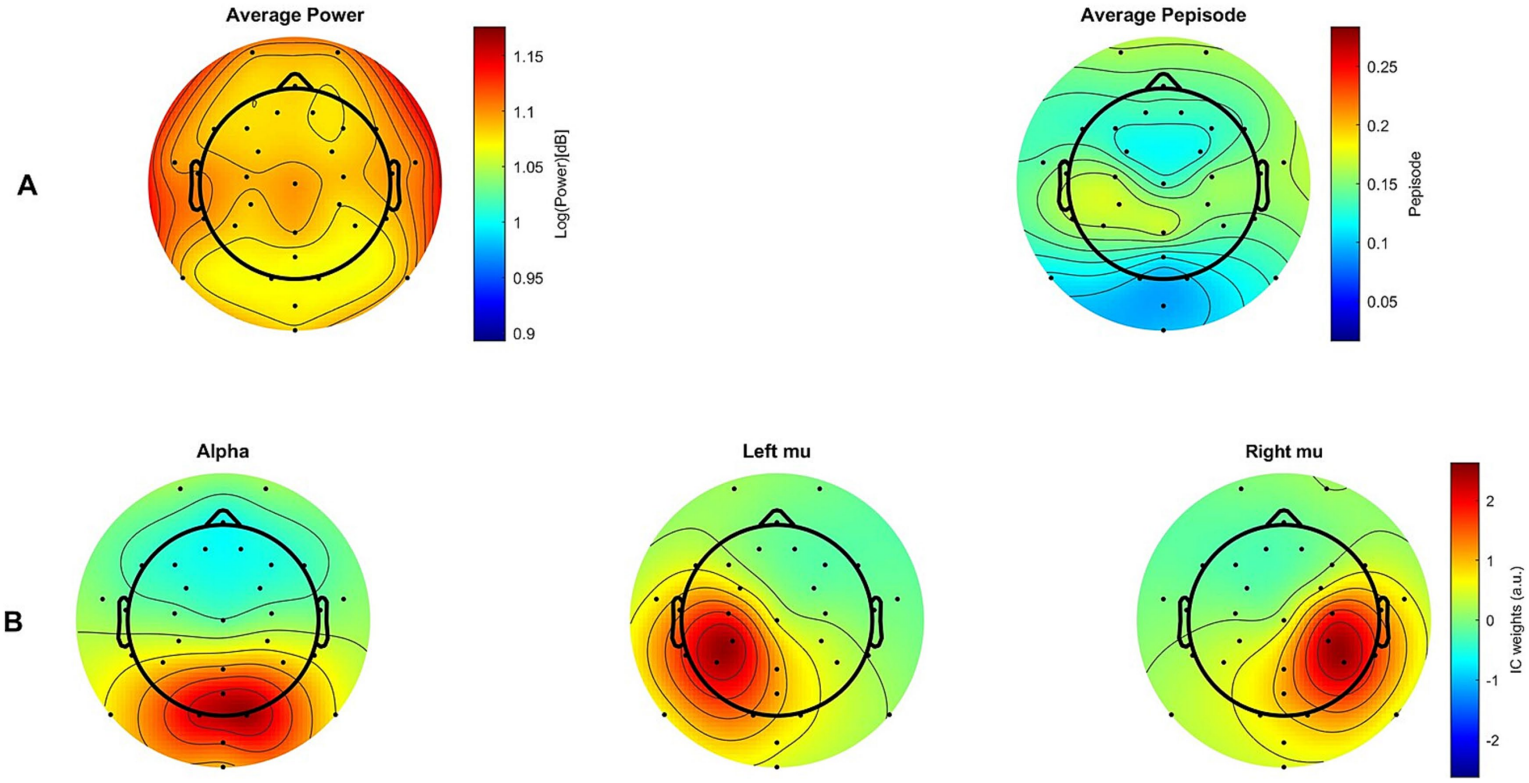
Topographies. **(A)** Maps showing group average power measures (left) and group average Pepisode measures (right) across all channels **(B)** Group average spatial patterns (maps) for alpha, as well as left and right mu components. Only maps in **(B)**, made using CORRMAP, are RMS-normalized.

To support the interpretation of our results, we applied the fBOSC method not only to the selected ICs but also across all channels and participants, which allowed us to generate the average power and Pepisode maps shown in Figure 9A. For comparison, Figure 9B also displays the topographies of the average alpha, right mu and left mu ICs constructed using CORRMAP (Campos Viola et al., 2009). CORRMAP identified ICs similar to user-defined templates based on alpha, mu and left ICs from subjects with the highest percentage of brain activity as determined by IClabel. Average maps are then generated from these clustered ICs, which may have excluded some participants from the 19 total.

Topographical maps of both Pepisode and power measures show their maximum activity in the sensorimotor area of the brain. However, the Pepisode map shows a more localized and pronounced concentration in this area compared to the power, which is more evenly distributed. The IC maps illustrate that the mu ICs likely captured most of the activity. Although no main effect of region was found, this corresponds to the observation that activity in the motor region (mu ICs) was higher compared to the visual region (alpha ICs), particularly for the Pepisode measure (cf. Figure 5).

#### 3.3.6 Effects of task repetition

To further examine effects of repeated exposure to TAMI items across the three blocks as well as possible impacts on neural dynamics, we conducted a series of rm-ANOVAs to assess the effect of block on all measures (TAMI score, Ease of Imagination score, oscillatory neural activity measures Pepisode and log power). This was done in motor and visual regions.

For behavioral measures (TAMI score and Ease of Imagination scale score) Bayesian analyses provided anecdotal evidence for the null hypothesis (BF_incl_ values between ⅓ and 1) indicating inconclusive results. In line with this, no significant block effects were found for either the TAMI or the Ease of Imagination scale score using frequentist statistics (all *p* > 0.05). In contrast, anecdotal to strong evidence supported a block effect on the motor-region oscillatory activity: BF_incl_ = 2.946 for Pepisode and BF_incl_ = 25.103 for power. Frequentist rm-ANOVAs mirrored these findings revealing significant effects of block for both Pepisode [*F* (2,36) = 4.488, *p* = 0.018] and power [*F*(2,36) = 5.455, *p* = 0.009]. Post-hoc analyses comparisons indicated that these effects were driven by differences between Block 1 and Block 3 for both Bayesian and frequentist tests. No evidence of an effect of block was observed for visual region oscillatory activity in either Bayesian (BF_incl_ values between 1 and 3) or frequentist analyses (all *p* > 0.05). Complete results and visualizations are provided in the Supplementary Figures S4, S5 and Supplementary Table S9.

#### 3.3.7 Self-reported motor imagery ability

To explore the role of self-reported motor imagery ability (visual or kinesthetic) on neural oscillatory patterns, we correlated the respective scores from the KVIQ (visual: *M* = 16.63, SD = 3.66; kinesthetic: *M* = 15.00, SD = 3.96) and MIQ questionnaires (internal visual: *M* = 5.33, SD = 0.85; external visual: *M* = 4.96, SD = 1.27; kinesthetic: *M* = 5.01, SD = 0.97) with the difference in mu and alpha oscillation suppression (successful – unsuccessful) during the TAMI, using both Pepisode and power measures. All correlations were weak to moderate in magnitude, with Bayesian analysis indicating anecdotal evidence, and *p*-values above the threshold of 0.05. The complete findings are detailed in Supplementary Table S11.

## 4 Discussion

In the present study, we investigated the relationship between mu and alpha oscillations and MI performance. Understanding how these oscillations and MI performance are related, both within and between individuals, may help to identify the neural correlates of performance variation, which is relevant to improving and extending the practical applicability of these rhythms. To ensure consistency, we followed Chen et al.’ (2021) work in using the TAMI to objectively measure MI performance, while implementing modifications to strengthen the methodology. First, at the intra-individual level, we hypothesized that there would be a greater decrease in mu/alpha activity for successful trials (i.e., correct responses) compared to unsuccessful trials. Second, we expected this suppression to be particularly significant for the first stages of the MI process. At the inter-individual level, we hypothesized two different outcomes. On the one hand, skilled imagers (i.e., participants with better scores on the TAMI) might show less overall mu/alpha oscillatory activity than their less skilled counterparts. Or perhaps, skilled imagers would show less reduction in mu/alpha activity due to their more efficient neural representation and reduced sensorimotor engagement.

Contrary to our expectations and previous findings by Chen et al. (2021), we did not find at least moderate evidence for a difference between successful and unsuccessful trials. The absence of difference in oscillatory activity as a function of performance, observed at the individual level, is in line with our results at the group level. However, we did find moderate to strong evidence for a decrease in mu/alpha-wave amplitude in the early stages, highlighting the importance of the initiation of imagery in neural activity, regardless of response success.

### 4.1 Intra- and inter-individual variability in mu and alpha rhythms

Both our within-subject and between-subject comparisons do not support the idea that mu/alpha oscillations serve as indicators of either inter-individual ability or intra-individual success in MI performance. Our results regarding MI ability are consistent with results by Chen et al. (2021), Gibson et al. (2014), and Di Nota et al. (2017). They also reported no significant differences in oscillatory activity based on participants’ familiarity or proficiency with the imagined actions. However, common patterns across participants do not always reflect the full neurophysiological reality (Wriessnegger et al., 2020). This is particularly true for mu and alpha rhythms, where variability is far from a hidden phenomenon.

Chen et al. (2021) attributed the lack of differences in oscillatory activity based on MI ability to the quantitative instability of the mu rhythms across groups (Blankertz et al., 2010; Tangwiriyasakul et al., 2013). Similarly, in a re-evaluation of their previous studies Wriessnegger et al. (2020) also pointed to the inter-subject variability in the alpha band, which could bias results and reduce sensitivity to detect true effects on performance (Haegens et al., 2014). In the field of MI brain computer interfaces, between-subject variability due to differences in motor learning and behavior, brain function and topography is a recognized challenge (Saha and Baumert, 2020).

Does the individual level provide a better reflection of performance? Chen et al. (2021) observed that reduced activity preceded correct responses in the TAMI, leading them to conclude that these oscillations could reflect individual success. However, our study found no evidence to support this observation. While Chen et al. (2021) presented each of the 10 TAMI questions only once, we repeated questions three times to increase the number of trials entering EEG analysis per participant, particularly for incorrect trials. This reduced the likelihood that differences (or lack thereof) were due to insufficient data points. Despite this, we did not find evidence for a difference between successful and unsuccessful trials.

As an exploratory analysis, we used repeated measures correlations to account for the fact that participants took the TAMI three times. This provided an additional estimate of the within-subject association by removing the between subject variability that could have masked a success effect. Even with this approach, the direction, and strength of the associations remained similar and not significant. This raises the possibility that, unlike what was suggested by Chen et al. (2021), mu and alpha oscillations may not index MI performance at the intra-individual level either, at least not reliably.

At the within-subject level, the literature shows mixed results (Ahn and Jun, 2015). For example, although Wriessnegger et al. (2020), found a high level of variability across groups, they also found that ERD/S values remained relatively consistent within individuals across conditions and time points. However, there is also literature reporting intra-individual variability. Performance inconsistency between sessions in the same subject has been observed in brain computer interface calibrations, as the classifier derived from the first session is rarely effective in subsequent sessions (Krauledat, 2008). Haegens et al. (2014) used magnetoencephalography (MEG) to investigate how alpha peak frequency differed across cognitive conditions and regions of interest within and between subjects. They found that while inter-individual variability exceeded intra-individual variability, alpha peak frequency in posterior regions increased with greater cognitive demands and engagement. This suggests that, when comparing and interpreting power values between conditions and making links to performance, power differences may be confounded with frequency shifts. This underscores the need to consider the operational range of the alpha rhythm — and arguably the mu rhythm — at both the inter- and intra-subject levels. The interactions between within- and between-subject factors are also important, as temporal variability within-subjects in EEG patterns has been found to contribute significantly to overall between-subjects variability, as seen in electrophysiological correlation patterns in an EEG-fMRI study (Meyer et al., 2013). In addition, long-term factors, such as age, gender, and environmental conditions, as well as short-term factors, such as sleep, diet, motivation, and focus on the task — which can fluctuate from trial to trial — can also have implications on the overall result (Daeglau et al., 2021).

### 4.2 Starting strong: onset of imagery reflected in mu and alpha suppression

We observed more pronounced mu and alpha oscillatory suppression during the initial stages of the MI process, as defined by the movement steps in a TAMI question. This finding replicates the results of Chen et al. (2021), who also found a significant decrease during the first step for both mu and alpha oscillations. However, in this study, this occurred independent of response accuracy.

Previous research on mu and alpha suppression has described more pronounced patterns at early stages. For instance, Llanos et al. (2013) found that visual stimuli used for motor planning activate mu-rhythm, inducing a short-lasting phase-locked mu-response and a persistent decrease of non-phase locked mu-rhythms. These rhythms were more marked when used for motor planning compared to passive observation, similarly for both real and imagined movements. Other studies have reported early mu ERD effects when participants view graspable objects in familiar orientations, suggesting rapid motor preparation triggered by visual cues (Kumar et al., 2013). When anticipating touch at specific body sites, greater mu desynchronization was linked to improved performance, cognitive speed and executive functioning (Weiss et al., 2020). Likewise, pre-stimulus reductions in alpha activity may reflect anticipation in response to a visual cue (Mathewson et al., 2009). After stimulus onset, alpha band power has been implicated in filtering incoming sensory information and maintaining relevant details in working memory (Woodman et al., 2022; Zanto and Gazzaley, 2009).

In this study, mu and alpha oscillations showed similar activity across all analyses. Chen et al. (2021) found a main effect of location, with greater reductions in oscillatory activity found in the motor region as opposed to the visual region. Given that similar decreases in alpha and mu were associated with MI success, the findings in the occipital region were interpreted as internal visual imagery facilitating the process of MI on the TAMI, a test that relies on visual strategies (Madan and Singhal, 2013). In contrast, we found no clear distinction between the two (i.e., inconclusive evidence for a main effect of region), despite disentangling the rhythms by ICA. In an effort to further clarify the distinct roles of mu and alpha oscillations, we leveraged our data set to examine whether imagery modality influenced neural activity. Specifically, we correlated participants’ self-reported MI scores (KVIQ and MIQ scales) with the mu and alpha oscillatory activity during the TAMI. However, all correlations provided only anecdotal evidence in support for the absence of a relationship between MI modality and oscillatory activity. The lack of distinct oscillatory patterns makes it difficult to determine the precise roles of visual and kinesthetic strategies in this task and consequently limits our interpretations of participants’ attentional and sensorimotor engagement.

It is important to note that we found evidence of more pronounced suppression only for the Pepisode measure, which, while characterizing the same brain oscillations, may have captured additional information not captured by power measures alone. Whereas wavelets do not distinguish between signal duration and amplitude, Pepisode excels at detecting variations in signal duration (van Vugt et al., 2007). Additionally, by using the BOSC method, we were able to control for aperiodic activity in neural oscillations, an approach advocated to enhance methodological precision in neural oscillation studies (Donoghue et al., 2022).

### 4.3 TAMI and ease of imagination as measures of MI ability

We used an objective measure of MI ability, the TAMI, together with our own exploratory subjective scale that assesses ease of imagination. Ease of imagination has previously been associated with MI ability, both behaviorally (MIQ-3; Williams et al., 2012) and neurophysiologically (Guillot and Collet, 2010; ter Horst et al., 2013). Consequently, we expected the TAMI scores to correlate with our Ease of Imagination scale. The correlation turned out to be relatively weak and mainly supported by anecdotal evidence.

Interestingly, both measures followed a similar trajectory across blocks: participants increased performance and also reported increase of imagination in blocks 1 and 2, followed by a decrease in both measurements for block 3. This pattern may reflect task adjustment during the initial blocks and fatigue for the final block, rather than a relationship between the two measures. However, given these changes were not supported by at least moderate evidence, we remain cautious with our interpretations.

One possible explanation for the weak association could be the exploratory nature of the Ease of Imagination scale design. Factors such as central tendency bias – as the scale was mostly skewed to the left by participants scoring toward the middle descriptions–might come into play. Alternatively, we might consider that TAMI and ease of imagination simply describe dimensions of MI performance that are not exactly the same. The TAMI provides a view of MI that was previously recognized by its authors and in the study by Chen et al. (2021) to be limited to visual modality imagery from an internal perspective and to response accuracy. Moreover, the TAMI is not a pure MI task, it includes reading instructions and memory retention. In contrast, ease of imagination may be more indicative of controllability (Guillot et al., 2010), which could be influenced by various psychological processes, such as working memory. These considerations also highlight the complexity of MI and of the TAMI itself.

### 4.4 Limitations

It is worth noting that our evidence of absence does not definitively indicate the absence of the success effect. Several points should be taken into consideration. First, our sample size was limited at both the participant and trial levels. To ensure a sufficient probability of detecting an effect, we employed a sequential Bayesian sampling method (Schönbrodt and Wagenmakers, 2018). However, the need to exclude some participants due to difficulties in identifying their components, reduced our sample size more than expected. Replication Bayes Factor results indicated that we gained hardly any evidence from the second batch of data (6 additional participants). Similarly, the number of trials was constrained. We increased the number of questions by repeating the TAMI 10 original questions three times.

While repeating the TAMI questions aimed to address a limitation in Chen et al. (2021), it may have introduced other unintended limitations. Although we found no clear evidence of a behavioral improvement across blocks that would indicate learning, there was a trend toward decreased performance in the final block (with anecdotal support). In contrast, the neural data showed moderate evidence of an increase in mean mu activity from block 1 to 3, which we interpret as a sign of sensorimotor disengagement. This possibly contributes to the decline in performance and serves as a potential confounding factor in the results (Neuper et al., 2006).

Regarding the experimental procedure, both Chen et al. (2021) and the present study, had participants inside a chamber, answering questions on a computer. In this study—and presumably also in the study by Chen et al. (2021) — participants remained seated while doing the TAMI, as opposed to standing as portrayed in the TAMI questions. This discrepancy may constrain participants’ ability to engage fully in the imagery task. This is important given that motor execution prior to MI has been found to influence neural activity, meaning that previous engagement in the position or movement could have facilitated the observation of brain activity (Allami et al., 2014; Pascual-Leone et al., 1995). Moreover, although the TAMI was designed to assess MI, it relies solely on the visual modality, which further hinders participant’s sensorimotor engagement with the task (Madan and Singhal, 2013).

As for the use of ICA, the ICs acted as a lens through which we defined and analyzed brain activity. However, this focused perspective may have caused us to miss part of the bigger picture. While descriptively (cf Figure 9), the mu components align well with the central-posterior regions displaying the largest Pepisode values, this correspondence is less evident for power. Neither Pepisode nor power show topographic peaks corresponding to the alpha component. While the areas with the highest power or Pepisode values cannot automatically be assumed to be the areas with the largest differences, we cannot rule out that the ICA approach contributed to not capturing the latter. However, this limitation applies to an even larger degree to channel-based analysis approaches, where, in effect, a single channel selected for analysis acts as a narrow spatial filter or lens.

Additionally, following Haegen’s et al. (2014) conclusions, using fixed alpha frequency bands might have biased our results against certain subjects whose peaks fall outside the predefined range, or against questions of the TAMI that shifted this peak. As a result, the MI performance effect could have been underestimated or missed entirely. This issue is an increasing concern in neural oscillation research, as peak frequency variability has been observed not only between and within subjects (Haegens et al., 2014), but also across cortical locations (Mahjoory et al., 2020), and within participants during a task (Benwell et al., 2019; Wutz et al., 2018).

In this regard, additional sources of variability in our study, —such as the inclusion of left-and mixed-handed participants, differences in prior engagement with motor-related activities like sports, music or video games (between subjects differences), and the possible sensorimotor disengagement observed in the third block (within participants differences within a task)—may have further contributed to inconsistencies in oscillatory responses. Moving forward, validating oscillation band definitions should be considered a necessary methodological step in future oscillation studies (Donoghue et al., 2022).

### 4.5 Future directions

The results of our analyses provide several directions for future research. A key limitation of the TAMI is the number of questions and narrow focus on visual MI. To provide more conclusive evidence regarding mu and alpha rhythms in MI performance, future MI performance measures could benefit from an expanded set of questions, incorporating a broader range of movements, outcomes, and modalities. Moreover, adaptive or staircased procedures borrowed from psychophysics could also enhance MI performance assessment. Adaptive methods focus on trials that provide the most information, improving efficiency and reducing the impact of ceiling and floor effects inherent in fixed-difficulty measures (Kingdom and Prins, 2016), such as the TAMI. This approach could provide a more balanced distribution of correct and incorrect trials, increasing statistical power and sensitivity to detect performance-related differences.

Finally, while replication of Chen et al.’s (2021) findings would have further validated the TAMI as an objective measure at the neurophysiological level, the discrepancies found in our study—which cannot be attributed to a single factor—highlight important considerations for the field. On one hand, our results contribute to the ongoing dialogue about how a universal and widely accepted protocol for the assessment of MI has yet to be established (Chepurova et al., 2022). Furthermore, our findings raise awareness about the importance of carefully examining and refining the methodological principles guiding study design and analysis, for meaningful interpretation of neural oscillations in relation to cognitive performance (Donoghue et al., 2022).

## 5 Conclusion

To conclude, our study extends the literature on mu and alpha oscillations and their relationship to MI performance. Despite previous findings suggesting an association at the individual level, our data provide evidence for a lack of association at both the intra- and inter-individual levels. While this does not rule out an association, it does highlight the need for a comprehensive assessment of these rhythms within and between subjects, as well as the exploration of factors that may influence their variability. In addition, our study supports the idea that the mu and alpha rhythm reflects the generation of the initial motor representation. A deeper understanding of this relationship could enhance the utility of mu and alpha rhythms in the development of tailored interventions and training programs for different users and sessions.

## Data Availability

The datasets presented in this study can be found in online repositories. The names of the repository/repositories and accession number(s) can be found in the article.

## Footnotes

^1^ https://osf.io/86e9b

^2^ https://osf.io/tqxub/

^3^ https://gitlab.com/mariapaulavillabona/mu-alpha-mi-performance

^4^The original pre-registered threshold of >90% was not met. In some participants, mu components were visually identified below the threshold of brain activity (never below 64.6%) and to maintain consistency across the majority of participants, selection was primarily based on expert visual inspection.

^5^ https://github.com/neurofractal/fBOSC
