# Supplementary material for: Investigating mu and alpha oscillations as indicators of intra-individual success and inter-individual ability in motor imagery performance
Source: Front Psychol. 2025 Jun 25;16:1598196. doi: 10.3389/fpsyg.2025.1598196 (PMC12239834; doi:10.3389/fpsyg.2025.1598196)
Supplement: Supplementary file 1 [file Supplementary_file_1.DOCX]

***Supplementary Material***

# Supplementary Figures and Tables

Supplementary Table S1 shows the subset of electrodes analyzed for all participants, along with their locations. Section 2 summarizes the statistical analyses. Table S2 presents the descriptive statistics for both the TAMI test and the Ease of Imagination scale scores. We also include supplementary information for both Bayesian (Section 2.1) and Frequentist (Section 2.2) analyses. This includes Figures S1 to S3 and Tables S3 to S5 for the Bayesian analyses, which describe hypothesis two and the corresponding models. Tables S6 to S8 present the same analyses for our main hypothesis within the Frequentist framework. Section 3 presents supplementary information from the exploratory analyses. Figures S4 and S5 show how all our measurements varied across blocks, with Table S9 and S10 providing the corresponding statistical analyses in both Bayesian and frequentists framework. Table S11 reports the comparison of neural dynamics with KVIQ and MIQ scales. Section 4 provides the wording, and the scale used for our Ease of Imagination scale in both German (4.1) and English (4.2) versions.

# Subset of Electrodes and Locations

**Table S1.** Number of electrodes and locations. Values are standardized to a Theta of 90° for the plane through Fpz, T7, T8, Oz.

| Name | Number | Theta | Phi |
| --- | --- | --- | --- |
| 1 | 1 | 0 | 0 |
| 2 | 2 | 46 | 41 |
| 3 | 3 | 46 | -25 |
| 4 | 4 | 46 | -90 |
| 5 | 5 | -46 | 25 |
| 6 | 6 | -46 | -41 |
| 7 | 7 | 92 | 90 |
| 8 | 8 | 92 | 34 |
| 9 | 9 | 92 | -21 |
| 10 | 10 | 92 | -76 |
| 11 | 11 | -92 | 76 |
| 12 | 12 | -92 | 21 |
| 13 | 13 | -92 | -34 |
| 14 | 14 | 115 | 10 |
| 15 | 15 | -115 | -10 |
| 16 | 16 | 138 | -40 |
| 17 | 17 | 138 | -90 |
| 18 | 18 | -138 | 40 |
| IO | 19 | -130 | -72 |
| ECG | 20 | -180 | 50 |
| 28 | 28 | 46 | 8 |
| 31 | 31 | -46 | 8 |
| 33 | 33 | 69 | 76 |
| 34 | 34 | 69 | 49 |
| 37 | 37 | 69 | -35 |
| 39 | 39 | 69 | -90 |
| 41 | 41 | -69 | 7 |
| 44 | 44 | -69 | -21 |
| 46 | 46 | 92 | 62 |
| 47 | 47 | 92 | 6 |
| 50 | 50 | -92 | 6 |
| 56 | 56 | 115 | -90 |
| Ref | Ref | 130 | 88 |
| Gnd | Gnd | 57 | 90 |

# Supplementary Statistics

# Descriptive Statistics

**Table S2.** Descriptives of TAMI and Ease of Imagination scale scores.

|  | | n | | Mean | | SD | | Skewness | | SE  of Skewness | | Shapiro-Wilk | | P-value of Shapiro-Wilk | | Minimum | | Maximum | |
| --- | --- | --- | --- | --- | --- | --- | --- | --- | --- | --- | --- | --- | --- | --- | --- | --- | --- | --- | --- |
| TAMI Score |  | 19 |  | 15.930 |  | 4.280 |  | -0.507 |  | 0.524 |  | 0.957 |  | 0.521 |  | 5.667 |  | 22.000 |  |
| Ease of Imagination scale Score |  | 19 |  | 43.474 |  | 6.824 |  | 1.076 |  | 0.524 |  | 0.880 |  | 0.021 |  | 35.000 |  | 58.667 |  |

**2.2 Bayesian Analyses**


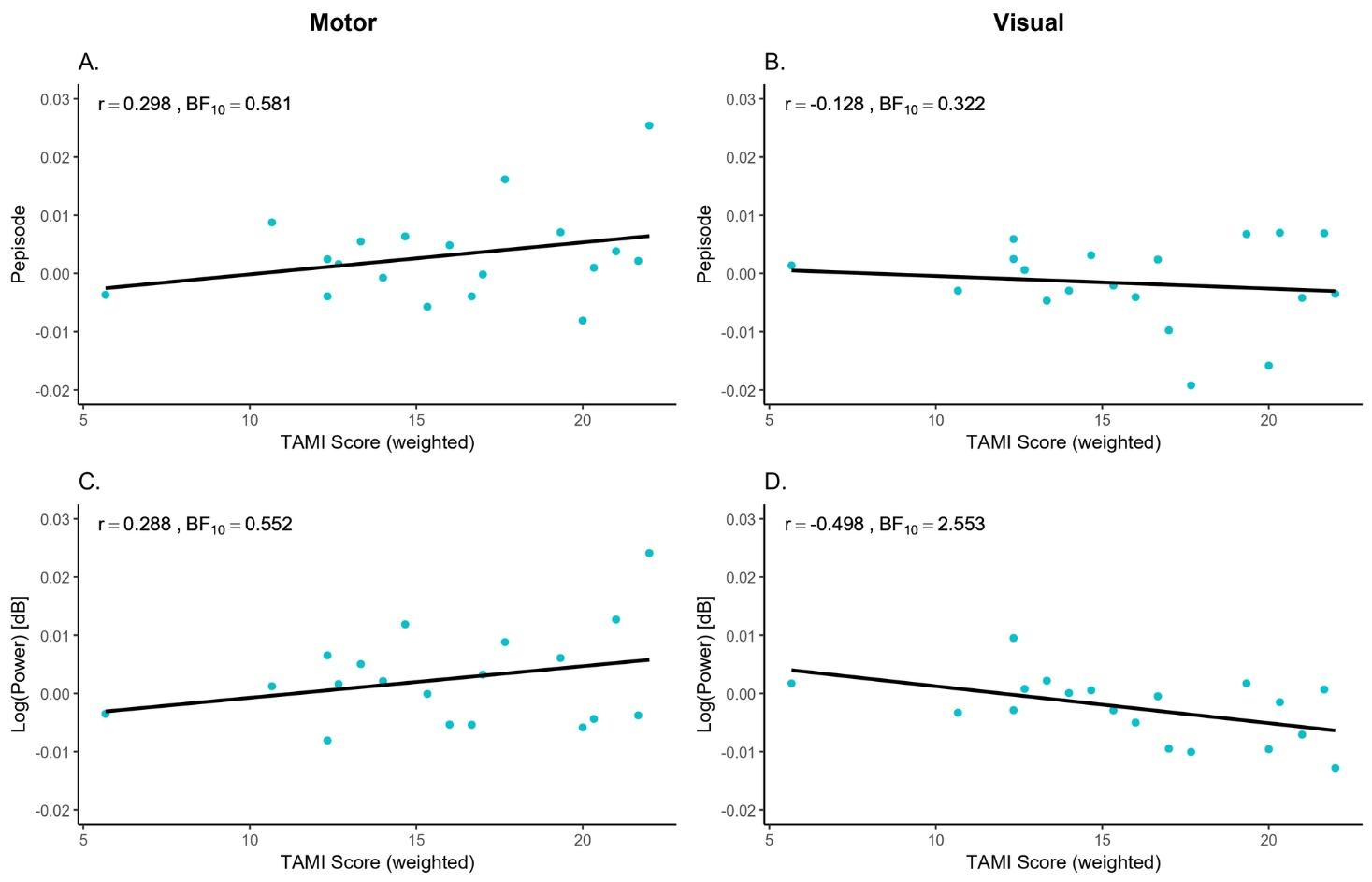


**Figure S1.** Motor imagery ability vs. TAMI scores. Scatter plots show the difference in motor imagery success (successful – unsuccessful trials) compared to their TAMI scores. Data is presented for the motor region: **(A)** Pepisode and **(C)** log power, and for the visual region: **(B)** Pepisode and **(D)** log power. Each dot represents one participant.


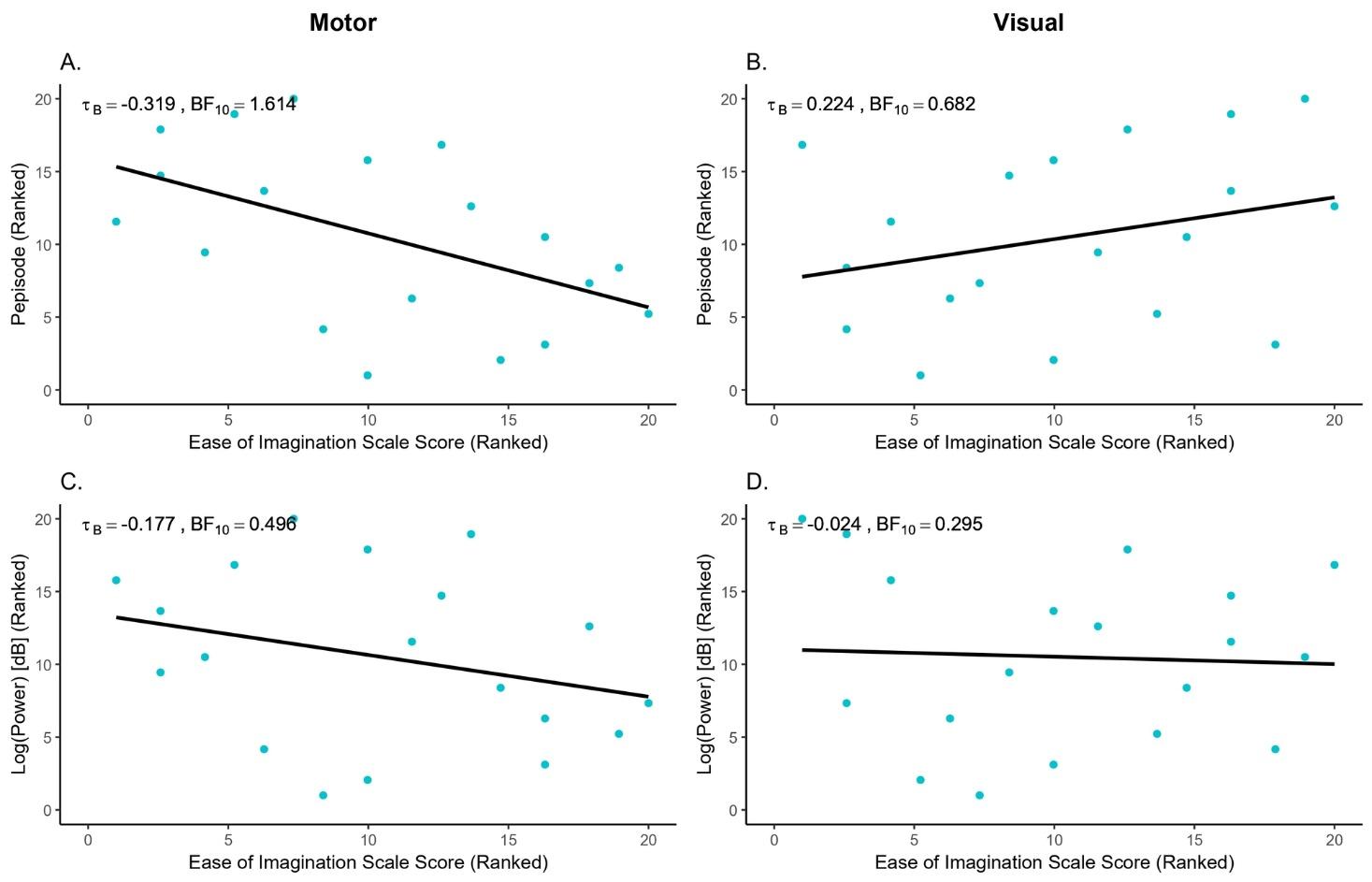


**Figure S2.** Motor imagery ability vs. Ease of Imagination scale scores. Scatter plots showing the difference in motor imagery success (successful – unsuccessful trials) compared to their Ease of Imagination scale scores. Data is presented for the motor region: **(A)** Pepisode and **(C)** log power, and for the visual region: **(B)** Pepisode and **(D)** log power. Each point represents an individual participant. For tau B, both the x- and y-axes represent ranked data.


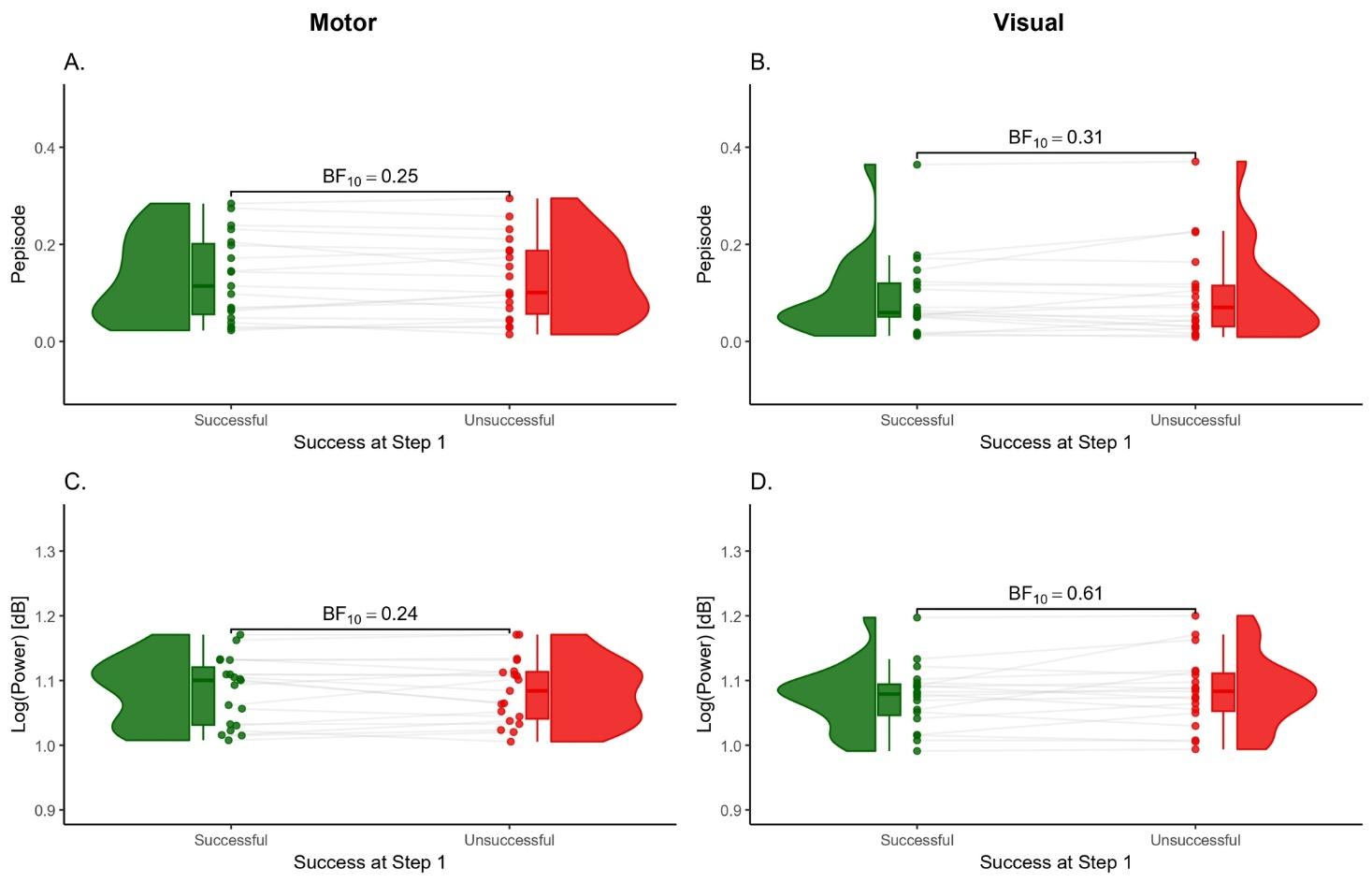


**Figure S3**.  Raincloud plots of success at step 1. Data is presented for the motor region: **(A)** Pepisode and **(C)** log power, and for the visual region: **(B)** Pepisode and **(D)** log power. Each dot represents one participant. Overlaid box plots indicate the interquartile range and median. The density distribution illustrates data spread.

**Table S3.** Model comparisons of Pepisode measures. The 'Model' column lists the predictors in each model. Adjacent columns show the prior and posterior model probabilities, posterior model odds and Bayes factors compared to the best model. The last column, 'error', estimates the numerical error in calculating the Bayes factor. These models are then sorted from lowest to highest Bayes factor.

| Model | | P(M) | | P(M\|data) | | BF_M_ | | BF_10_ | | error % | |
| --- | --- | --- | --- | --- | --- | --- | --- | --- | --- | --- | --- |
| Step |  | 0.053 |  | 0.303 |  | 7.815 |  | 1.000 |  |  |  |
| Region + Step |  | 0.053 |  | 0.252 |  | 6.077 |  | 0.834 |  | 24.939 |  |
| Success + Step |  | 0.053 |  | 0.095 |  | 1.880 |  | 0.312 |  | 3.856 |  |
| Null Model (Incl. Subject and Random Slopes) |  | 0.053 |  | 0.066 |  | 1.271 |  | 0.218 |  | 2.327 |  |
| Region + Step + Region ✻  Step |  | 0.053 |  | 0.055 |  | 1.041 |  | 0.181 |  | 6.061 |  |
| Success + Region + Step + Success ✻  Region |  | 0.053 |  | 0.055 |  | 1.040 |  | 0.180 |  | 35.568 |  |
| Success + Region + Step |  | 0.053 |  | 0.043 |  | 0.812 |  | 0.143 |  | 8.619 |  |
| Region |  | 0.053 |  | 0.037 |  | 0.695 |  | 0.123 |  | 19.787 |  |
| Success |  | 0.053 |  | 0.021 |  | 0.392 |  | 0.070 |  | 4.578 |  |
| Success + Region + Step + Region ✻  Step |  | 0.053 |  | 0.303 |  | 7.815 |  | 1.000 |  |  |  |

**Table S4.** Model comparisons of log power [dB] measures. The 'Model' column lists the predictors in each model. Adjacent columns show, respectively, the prior and posterior model probabilities, posterior model odds and Bayes factors compared to the best model. The last column, 'error', estimates the numerical error in calculating the Bayes factor. These models are then sorted from lowest to highest Bayes factor.

| Model | | P(M) | | P(M\|data) | | BF_M_ | | BF_10_ | | error % | |
| --- | --- | --- | --- | --- | --- | --- | --- | --- | --- | --- | --- |
| Null Model (Incl. Subject and Random Slopes) |  | 0.053 |  | 0.238 |  | 5.631 |  | 1.000 |  |  |  |
| Step |  | 0.053 |  | 0.180 |  | 3.944 |  | 0.754 |  | 2.283 |  |
| Region + Step |  | 0.053 |  | 0.169 |  | 3.655 |  | 0.708 |  | 18.383 |  |
| Region |  | 0.053 |  | 0.157 |  | 3.349 |  | 0.658 |  | 2.266 |  |
| Region + Step + Region ✻  Step |  | 0.053 |  | 0.051 |  | 0.977 |  | 0.216 |  | 8.438 |  |
| Success |  | 0.053 |  | 0.046 |  | 0.863 |  | 0.192 |  | 3.751 |  |
| Success + Region |  | 0.053 |  | 0.037 |  | 0.692 |  | 0.155 |  | 10.967 |  |
| Success + Step |  | 0.053 |  | 0.033 |  | 0.607 |  | 0.137 |  | 2.089 |  |
| Success + Region + Step |  | 0.053 |  | 0.028 |  | 0.526 |  | 0.119 |  | 11.474 |  |
| Success + Region + Success ✻  Region |  | 0.053 |  | 0.018 |  | 0.336 |  | 0.077 |  | 7.729 |  |

**Table S5.** Parameter estimates for each of the steps. Posteriors are summarized using mean, standard deviation, and 95% central credible intervals (CI).

|  | | | | | | | | 95% Credible Interval | | | |
| --- | --- | --- | --- | --- | --- | --- | --- | --- | --- | --- | --- |
| Predictor | | Level | | Mean | | SD | | Lower | | Upper | |
| Intercept |  |  |  | 0.128 |  | 0.018 |  | 0.090 |  | 0.163 |  |
| Step |  | Step 1 |  | -0.012 |  | 0.005 |  | -0.024 |  | -0.003 |  |
|  |  | Step 2 |  | 0.002 |  | 0.005 |  | -0.007 |  | 0.011 |  |
|  |  | Step 3 |  | 0.005 |  | 0.005 |  | -0.005 |  | 0.013 |  |
|  |  | Step 4 |  | 0.005 |  | 0.005 |  | -0.006 |  | 0.012 |  |

**2.3. Frequentist Analyses**

**Table S6.** Frequentist within-participant effects with sphericity corrections for Pepisode. ᵃ Mauchly's test of sphericity indicates that the assumption of sphericity is violated (p < .05). *p-values ≤ 0.05.

| Cases | Sphericity Correction | Sum of Squares | df | Mean Square | F | p |
| --- | --- | --- | --- | --- | --- | --- |
| Success | — | 9.295×10⁻⁴ | 1.000 | 9.295×10⁻⁴ | 2.076 | 0.167 |
| Residuals | — | 0.008 | 18.000 | 4.478×10⁻⁴ |  |  |
|  |  |  |  |  |  |  |
| Region | — | 0.187 | 1.000 | 0.187 | 2.607 | 0.124 |
| Residuals | — | 1.294 | 18.000 | 0.072 |  |  |
|  |  |  |  |  |  |  |
| Step | — | 0.028ᵃ | 3.000ᵃ | 0.009ᵃ | 3.961ᵃ | 0.013ᵃ* |
|  | Greenhouse-Geisser | 0.028 | 1.884 | 0.015 | 3.961 | 0.031* |
| Residuals | — | 0.126 | 54.000 | 0.002 |  |  |
|  | Greenhouse-Geisser | 0.126 | 33.916 | 0.004 |  |  |
|  |  |  |  |  |  |  |
| Success ✻ Region | — | 0.002 | 1.000 | 0.002 | 2.684 | 0.119 |
| Residuals | — | 0.016 | 18.000 | 8.715×10⁻⁴ |  |  |
|  |  |  |  |  |  |  |
| Success ✻ Step | — | 0.001 | 3.000 | 3.984×10⁻⁴ | 0.721 | 0.544 |
|  | Greenhouse-Geisser | 0.001 | 2.059 | 5.805×10⁻⁴ | 0.721 | 0.497 |
| Residuals | — | 0.030 | 54.000 | 5.526×10⁻⁴ |  |  |
|  | Greenhouse-Geisser | 0.030 | 37.058 | 8.052×10⁻⁴ |  |  |
|  |  |  |  |  |  |  |
| Region ✻ Step | — | 0.006ᵃ | 3.000ᵃ | 0.002ᵃ | 1.297ᵃ | 0.285ᵃ |
|  | Greenhouse-Geisser | 0.006 | 1.871 | 0.003 | 1.297 | 0.285 |
| Residuals | — | 0.087 | 54.000 | 0.002 |  |  |
|  | Greenhouse-Geisser | 0.087 | 33.680 | 0.003 |  |  |
|  |  |  |  |  |  |  |
| Success ✻ Region ✻ Step | — | 4.471×10⁻⁴ | 3.000 | 1.490×10⁻⁴ | 0.318 | 0.813 |
|  | Greenhouse-Geisser | 4.471×10⁻⁴ | 2.344 | 1.907×10⁻⁴ | 0.318 | 0.763 |
| Residuals | — | 0.025 | 54.000 | 4.693×10⁻⁴ |  |  |
|  | Greenhouse-Geisser | 0.025 | 42.201 | 6.006×10⁻⁴ |  |  |

**Table S7.** Frequentist post hoc tests for step factors from Pepisode measures. *p-values ≤ 0.05. P-value adjusted for comparing a family of 6.

|  | |  | | Mean Difference | | SE | | t | | Cohen's d | | p_bonf_ | |
| --- | --- | --- | --- | --- | --- | --- | --- | --- | --- | --- | --- | --- | --- |
| Step 1 |  | Step 2 |  | -0.020 |  | 0.008 |  | -2.509 |  | -0.186 |  | 0.091 |  |
|  |  | Step 3 |  | -0.023 |  | 0.008 |  | -2.979 |  | -0.221 |  | 0.026 | * |
|  |  | Step 4 |  | -0.023 |  | 0.008 |  | -2.869 |  | -0.213 |  | 0.035 | * |
| Step 2 |  | Step 3 |  | -0.004 |  | 0.008 |  | -0.470 |  | -0.035 |  | 1.000 |  |
|  |  | Step 4 |  | -0.003 |  | 0.008 |  | -0.360 |  | -0.027 |  | 1.000 |  |
| Step 3 |  | Step 4 |  | 8.669×10^-4^ |  | 0.008 |  | 0.110 |  | 0.008 |  | 1.000 |  |

**Table S8.** Frequentist within-participant effects with sphericity corrections for log power [dB]. ᵃ Mauchly's test of sphericity indicates that the assumption of sphericity is violated (p < .05). *p-values ≤ 0.05.

| Cases | Sphericity Correction | Sum of Squares | df | Mean Square | F | p |
| --- | --- | --- | --- | --- | --- | --- |
| Success | — | 5.295×10⁻⁵ | 1.000 | 5.295×10⁻⁵ | 0.245 | 0.626 |
| Residuals | — | 0.004 | 18.000 | 2.158×10⁻⁴ |  |  |
|  |  |  |  |  |  |  |
| Region | — | 0.014 | 1.000 | 0.014 | 0.932 | 0.347 |
| Residuals | — | 0.275 | 18.000 | 0.015 |  |  |
|  |  |  |  |  |  |  |
| Step | — | 0.006ᵃ | 3.000ᵃ | 0.002ᵃ | 2.277ᵃ | 0.090ᵃ |
|  | Greenhouse-Geisser | 0.006 | 1.791 | 0.003 | 2.277 | 0.124 |
| Residuals | — | 0.044 | 54.000 | 8.124×10⁻⁴ |  |  |
|  | Greenhouse-Geisser | 0.044 | 32.240 | 0.001 |  |  |
|  |  |  |  |  |  |  |
| Success ✻ Region | — | 8.046×10⁻⁴ | 1.000 | 8.046×10⁻⁴ | 2.151 | 0.160 |
| Residuals | — | 0.007 | 18.000 | 3.740×10⁻⁴ |  |  |
|  |  |  |  |  |  |  |
| Success ✻ Step | — | 0.001 | 3.000 | 3.446×10⁻⁴ | 1.066 | 0.371 |
|  | Greenhouse-Geisser | 0.001 | 2.418 | 4.276×10⁻⁴ | 1.066 | 0.363 |
| Residuals | — | 0.017 | 54.000 | 3.233×10⁻⁴ |  |  |
|  | Greenhouse-Geisser | 0.017 | 43.519 | 4.012×10⁻⁴ |  |  |
|  |  |  |  |  |  |  |
| Region ✻ Step | — | 0.002ᵃ | 3.000ᵃ | 7.881×10⁻⁴ᵃ | 1.360ᵃ | 0.265ᵃ |
|  | Greenhouse-Geisser | 0.002 | 1.780 | 0.001 | 1.360 | 0.269 |
| Residuals | — | 0.031 | 54.000 | 5.796×10⁻⁴ |  |  |
|  | Greenhouse-Geisser | 0.031 | 32.047 | 9.766×10⁻⁴ |  |  |
|  |  |  |  |  |  |  |
| Success ✻ Region ✻ Step | — | 5.470×10⁻⁴ | 3.000 | 1.823×10⁻⁴ | 0.693 | 0.560 |
|  | Greenhouse-Geisser | 5.470×10⁻⁴ | 2.467 | 2.217×10⁻⁴ | 0.693 | 0.534 |
| Residuals | — | 0.014 | 54.000 | 2.630×10⁻⁴ |  |  |
|  | Greenhouse-Geisser | 0.014 | 44.403 | 3.198×10⁻⁴ |  |  |

# 3. Exploratory Supplementary Material


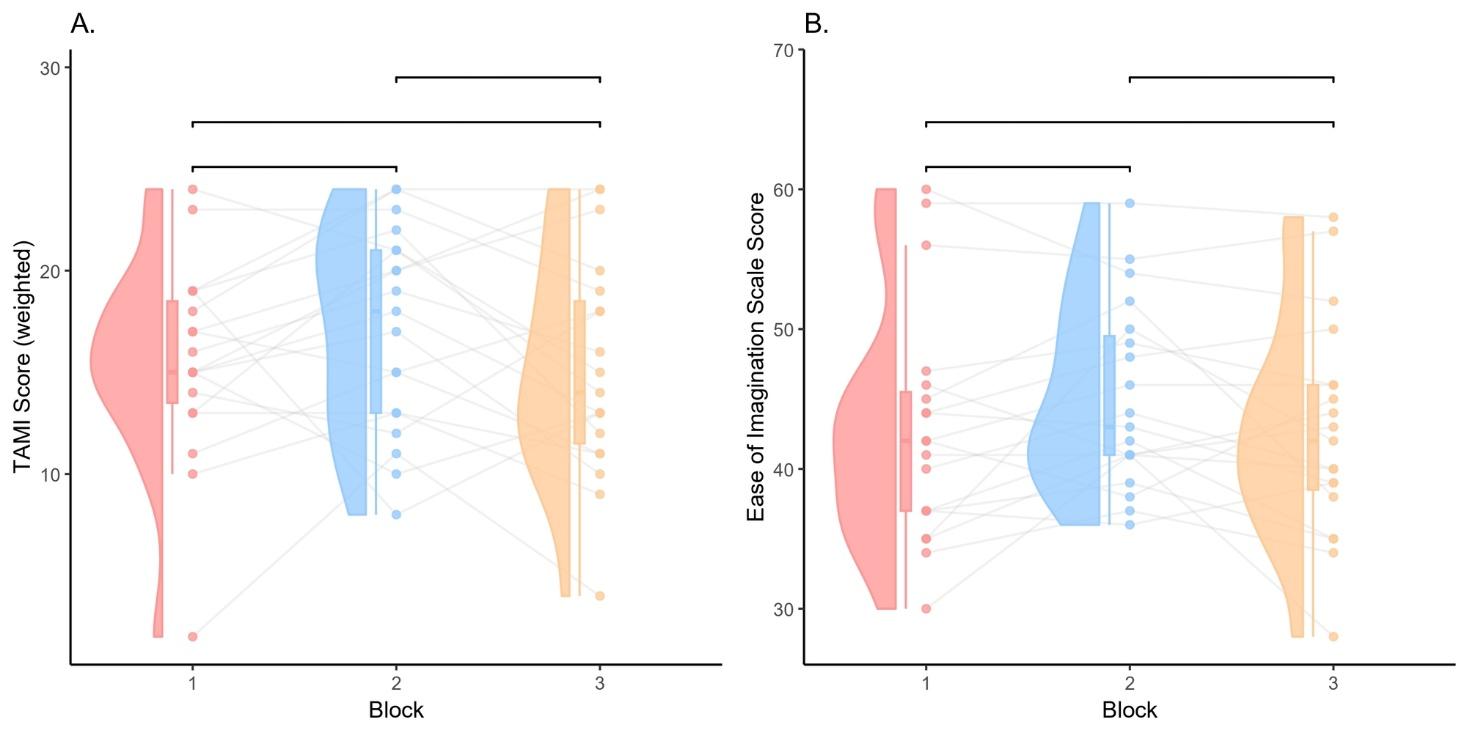


**Figure S4**.  Score variations across blocks for **(A)** the TAMI, and **(B)** the Ease of Imagination scale. Each dot represents one participant. Overlaid box plots indicate the interquartile range and median. The density distribution illustrates data spread Unmarked comparisons indicate inconclusive/null evidence Bayes Factors (BF₁₀) evidence (details in Table S9 and S10).


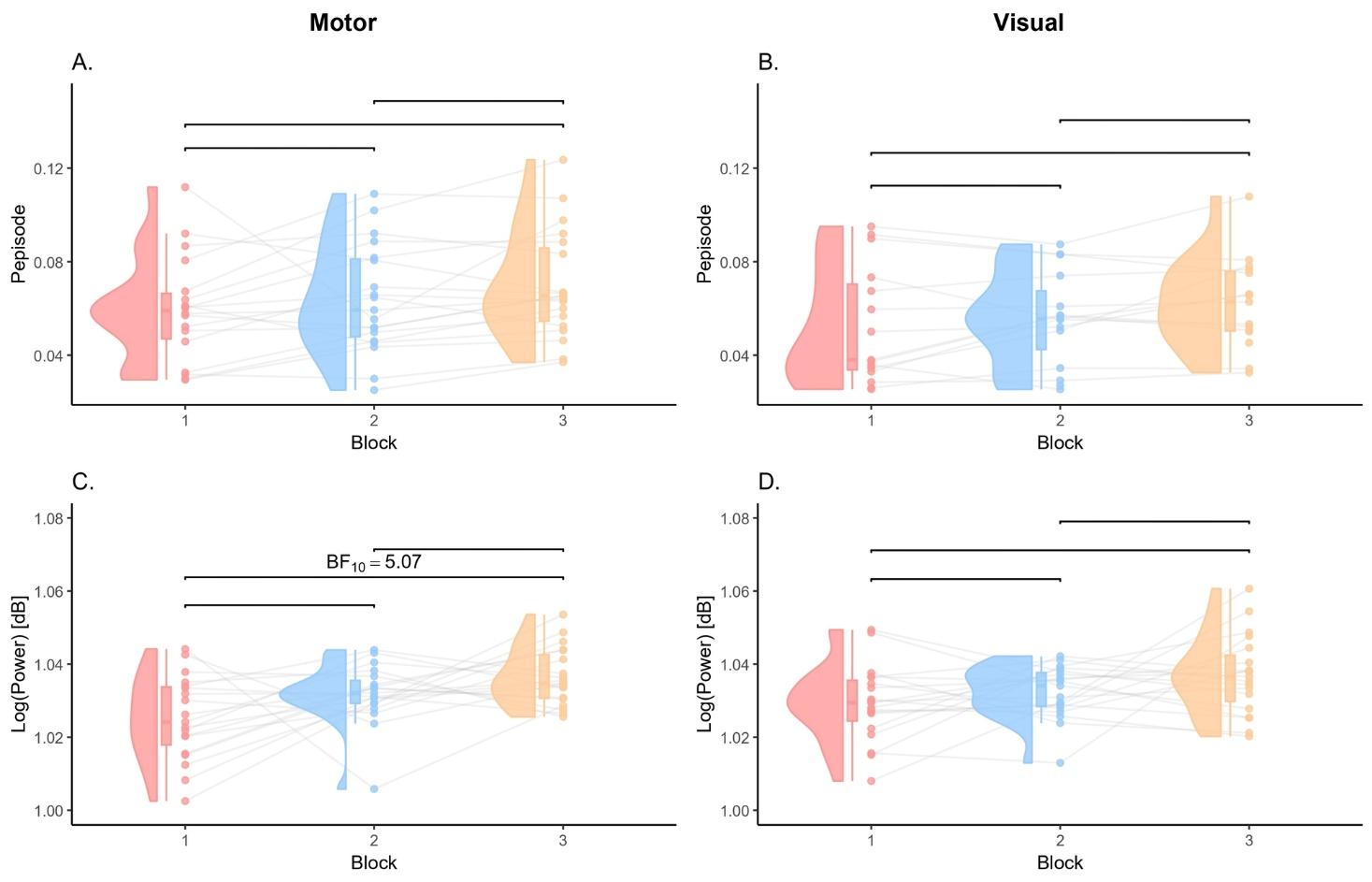
**Figure S5**.  Neural dynamics variations across blocks. Data is presented for the motor region: **(A)** Pepisode and **(C)** log power, and for the visual region: **(B)** Pepisode and **(D)** log power. Each dot represents one participant. Overlaid box plots indicate the interquartile range and median. Unmarked comparisons indicate inconclusive/null evidence Bayes Factors (BF₁₀) evidence. See Table S9 and S10 for full statistical details.

**Table S9.** Bayesian within-participants results for the effect of block. For post-hoc comparisons, only those with substantial evidence (BF₁₀,U > 3) are listed.

| Measure | With Subjects Effect | BF_incl_ | Evidence | Significant Post-Hoc Comparisons | Post-Hoc BF₁₀,U |
| --- | --- | --- | --- | --- | --- |
| TAMI | Block | 0.576 | Anecdotal evidence for H₀ | None | - |
| Ease of Imagination scale |  | 0.640 | Anecdotal evidence for H₀ | None | - |
| Pepisode Alpha |  | 1.288 | Anecdotal evidence for H₁ | None | - |
| Power Alpha |  | 1.441 | Anecdotal evidence for H₁ | None | - |
| Pepisode Mu |  | 2.946 | Anecdotal evidence for H₁ | None | - |
| Power Mu |  | 25.103 | Strong evidence for H₁ | Block 1 < Block 3 | 5.070 |

**Table S10.** Frequentist within-participant effects for the effect of block. For measures with Greenhouse-Geisser correction, Mauchly's test indicated that the assumption of sphericity was violated (p < .05). Post-hoc comparisons used Bonferroni correction for multiple comparisons. All analyses used Type III Sum of Squares. Significant results (p < .05) are marked with an asterisk (*).

| Measure | Within Subjects Effects | Sphericity Correction | Sum of Squares | df | Mean Square | F | p | Significant Post-Hoc Comparisons |
| --- | --- | --- | --- | --- | --- | --- | --- | --- |
| TAMI | Block | — | 44.667 | 2 | 22.333 | 2.000 | 0.150 | — |
| Ease of Imagination scale |  | — | 71.053 | 2 | 35.526 | 2.145 | 0.132 | — |
| Pepisode Alpha |  | Greenhouse-Geisser | 5.660×10⁻⁴ | 1.526 | 3.709×10⁻⁴ | 3.223 | 0.067 | — |
| Power Alpha |  | Greenhouse-Geisser | 5.600×10⁻⁴ | 1.406 | 3.983×10⁻⁴ | 2.453 | 0.120 | — |
| Pepisode Mu |  | — | 0.001 | 2 | 7.020×10⁻⁴ | 4.488 | 0.018* | Block 1 < Block 3 (p = 0.015) |
| Power Mu |  | — | 0.001 | 2 | 6.122×10⁻⁴ | 5.455 | 0.009* | Block 1 < Block 3 (p = 0.007) |

**Table S11**. Bayesian and frequentist correlations for relationships between mu and alpha oscillatory activity and self-reported motor imagery ability. KVIQ and MIQ scales are compared with the Pepisode and log power [dB] values. Pepisode and log power values indicate the difference between successful and unsuccessful trials on the TAMI and Ease of Imagination scale scores. Scores are averaged over the three blocks per participant.

| Imagery Measure | Power mu | | | Power alpha | | | Pepisode mu | | | Pepisode alpha | | |
| --- | --- | --- | --- | --- | --- | --- | --- | --- | --- | --- | --- | --- |
|  | Pearson's r | p-value | BF₁₀ | Pearson's r | p-value | BF₁₀ | Pearson's r | p-value | BF₁₀ | Pearson's r | p-value | BF₁₀ |
| KVIQ visual | 0.334 | 0.162 | 0.707 | 0.121 | 0.621 | 0.318 | 0.070 | 0.776 | 0.295 | 0.253 | 0.296 | 0.472 |
| KVIQ kinesthetic | -0.254 | 0.295 | 0.474 | 0.207 | 0.395 | 0.398 | -0.148 | 0.546 | 0.336 | 0.075 | 0.760 | 0.296 |
| MIQ internal visual | -0.025 | 0.921 | 0.285 | 0.247 | 0.309 | 0.460 | 0.031 | 0.899 | 0.286 | 0.398 | 0.092 | 1.067 |
| MIQ external visual | -0.030 | 0.902 | 0.286 | 0.133 | 0.587 | 0.326 | -0.026 | 0.917 | 0.285 | -0.013 | 0.957 | 0.284 |
| MIQ kinesthetic | -0.007 | 0.977 | 0.284 | 0.072 | 0.769 | 0.295 | 0.058 | 0.815 | 0.291 | 0.052 | 0.834 | 0.290 |

# 4. Ease of Imagination Scale: German and English Versions

**4.1 German**

Bitte bewerten Sie den Grad der Leichtigkeit oder Schwierigkeit, den Sie bei der Bewältigung der Aufgabe empfunden haben.

Verwenden Sie die folgende Skala, um Ihre Bewertung anzugeben, indem Sie die entsprechende Zahl auf der Tastatur drücken.

**Skala:** 1 = „Sehr schwer“, 2 = „Schwer“, 3 = „Etwas schwer“, 4 = „Neutral (nicht leicht/nicht schwer)“, 5 = „Etwas leicht“, 6 = „Leicht“ und 7 = „Sehr leicht“

**4.2 English**

Please rate the level of ease or difficulty you experienced while completing the task.

Use the following scale to indicate your rating by pressing the corresponding number on the keyboard.

**Scale:** 1 = "Very hard," 2 = "Hard," 3 = "Somewhat hard," 4 = "Neutral (not easy, not hard)," 5 = "Somewhat easy," 6 = "Easy," and 7 = "Very easy."
